# Music production and its role in coalition signaling during foraging contexts in a hunter-gatherer society

**DOI:** 10.3389/fpsyg.2023.1218394

**Published:** 2023-11-01

**Authors:** Chirag Rajendra Chittar, Haneul Jang, Liran Samuni, Jerome Lewis, Henkjan Honing, E. Emiel van Loon, Karline R. L. Janmaat

**Affiliations:** ^1^Institute for Biodiversity and Ecosystem Dynamics (IBED), University of Amsterdam, Amsterdam, Netherlands; ^2^Department of Evolutionary Anthropology, University of Zurich, Zurich, Switzerland; ^3^Department of Human Behavior, Ecology and Culture, Max Planck Institute for Evolutionary Anthropology, Leipzig, Germany; ^4^Department of Human Evolutionary Biology, Harvard University, Cambridge, MA, United States; ^5^Cooperative Evolution Lab, German Primate Center, Göttingen, Germany; ^6^Department of Anthropology, University College London, London, United Kingdom; ^7^Music Cognition Group, Institute for Logic, Language and Computation, University of Amsterdam, Amsterdam, Netherlands; ^8^Department of Cognitive Psychology, Leiden University, Leiden, Netherlands; ^9^ARTIS Amsterdam Royal Zoo, Amsterdam, Netherlands

**Keywords:** music, foraging, coalition signaling, less familiar, predation, hunter-gatherer

## Abstract

Music is a cultural activity universally present in all human societies. Several hypotheses have been formulated to understand the possible origins of music and the reasons for its emergence. Here, we test two hypotheses: (1) the coalition signaling hypothesis which posits that music could have emerged as a tool to signal cooperative intent and signal strength of alliances and (2) music as a strategy to deter potential predators. In addition, we further explore the link between tactile cues and the propensity of mothers to sing toward infants. For this, we investigated the singing behaviors of hunter-gatherer mothers during daily foraging trips among the Mbendjele BaYaka in the Republic of the Congo. Although singing is a significant component of their daily activities, such as when walking in the forest or collecting food sources, studies on human music production in hunter-gatherer societies are mostly conducted during their ritual ceremonies. In this study, we collected foraging and singing behavioral data of mothers by using focal follows of five BaYaka women during their foraging trips in the forest. In accordance with our predictions for the coalition signaling hypothesis, women were more likely to sing when present in large groups, especially when group members were less familiar. However, predictions of the predation deterrence hypothesis were not supported as the interaction between group size and distance from the village did not have a significant effect on the likelihood of singing. The latter may be due to limited variation in predation risk in the foraging areas, because of the intense bush meat trade, and hence, future studies should include foraging areas with higher densities of wild animals. Lastly, we found that mothers were more likely to sing when they were carrying infants compared to when infants were close, but carried by others, supporting the prediction that touch plays an important prerequisite role in musical interaction between the mother and child. Our study provides important insight into the role of music as a tool in displaying the intent between or within groups to strengthen potentially conflict-free alliances during joint foraging activities.

## Introduction

Music is a ubiquitous cultural feature that is present wherever human beings are found (Brown, 1991; Savage et al., 2015; Trehub et al., 2015; Mehr et al., 2019) and has been actively studied in an attempt to deconstruct its physiological, neurological, psychological, genetic, developmental, and cultural impacts on humans (Wallin et al., 2000; Honing, 2018). Theories on the origins and evolution of music have been diverse and plenty (Huron, 2001), although they are regularly debated (Bloom and Finlay, 2021; Honing, 2021, 2023). One of the earliest approaches to explain the evolution of music was taken by Darwin (1871) who compared music to mating calls and suggested that music could have arisen as an adaptation through sexual selection allowing early men to compete with each other to court and win over women. However, the sexual selection hypothesis fails to explain the variety of contexts in which music takes place such as war rituals, work, mourning, and social gatherings, as well as the presence of music in children and the listening by people of all ages (Hagen and Bryant, 2003; Dissanayake, 2006; Clayton, 2009; Kirschner and Tomasello, 2010; Mehr et al., 2019, 2021).

As a result, another new hypothesis was put forward to explain the occurrence of music in social groups, stating that music could have originated to initiate and enhance social bonding within and between groups: the social bonding hypothesis (Roederer, 1984; Savage et al., 2021). Novel social bonds and bond strength are crucial for humans and non-human primates as they are suggested to positively affect longevity, mental wellbeing, access to nutritionally rich but difficult-to-acquire food, dominance rank, territorial defense and expansion, predator protection, and reproductive success (Kaplan et al., 2000; Silk, 2007; Dunbar and Shultz, 2010; Holt-Lunstad et al., 2010; Silk et al., 2010; Dunbar, 2012; Gilby et al., 2013; Samuni et al., 2018). According to the social bonding hypothesis, music is thought to achieve increased social bonding by facilitating synchronization between and among individuals in groups (Demos et al., 2012; Weinstein et al., 2016; Savage et al., 2021).

However, as music is largely uninformative concerning personal goals, skills, and fitness benefits of individuals (an important outcome of social bonding), it may hinder reciprocal exchange (Hagen and Bryant, 2003). In addition, Mehr et al. (2021) suggested that music production is costly due to its energy and time-intensive nature. Hence, an alternative to the social bonding hypothesis has been raised where music does not cause bonding and boost cohesion directly but can reliably signal coalition strength or quality and cooperative intent or the intention to signal information on the willingness to cooperate within and between groups (Hagen and Bryant, 2003; Mehr et al., 2021). According to Mehr et al. (2021), the coalition signaling hypothesis is part of a larger hypothesis (the credible signaling hypothesis), where music could act as a reliable signal to deter predation, signal attention toward altricial infants, and signal coalition strength or intent. Therefore, music in group contexts could signal group identity, coalition strength, or quality through showcasing complex coordinated or synchronized musical performances for spectating groups and, hence, also displaying a group identity through celebration or remembrance of ancestors (McCall, 1993; Feld, 2012; Mehr et al., 2021). Furthermore, music could be used as an intimidation strategy in inter-group encounters (Zavos, 1998; Jordania, 2015). At the same time, music under certain contexts has been argued to signal information regarding the intention to form and strengthen coalitions by “breaking the ice” between individuals from different groups or within groups and in turn indirectly benefitting group members by giving potential future access to valuable information regarding resources. Feasts, rituals, and processions are major social contexts that are largely accompanied by music to encourage new alliances and exhibit group identities and cooperation across several cultures (Zvelebil, 1988; Hagen and Bryant, 2003; Levitin, 2008; Ng and Verkuyten, 2010; Boer and Abubakar, 2014; Hayden, 2014; Lewis, 2015; Mehr et al., 2021). For example, among the Columbian Barasana, food is donated by one of the groups to other neighboring local groups as an act of gratitude and as an incentive to build trust. The period of food exchange is accompanied by music and dance throughout the entire day and night (Hugh-Jones, 1979). Furthermore, the number of individuals singing and dancing, the extent of coordination, or the amount of time invested in practicing could signify the eagerness to achieve a particular group-benefitting goal (Rouget, 1980; Hagen and Bryant, 2003; Mehr et al., 2021). Although the role of music in signaling coalitions has been thoroughly formulated (Mehr et al., 2021), to our knowledge, it has not been empirically tested yet on a non-WEIRD (Western, Educated, Industrialized, Rich, and Democratic; Henrich et al., 2010) population. This current article is one of the first attempts to do so.

Another argument under the credible signaling hypothesis posits that music originated as a strategy along with the use of masks, body painting, and clothes to enable a group to intimidate and deter threats, such as predators (Jordania, 2011; Hagen, 2022). Singing in large numbers is shown to have a positive effect on singing amplitude, although conditional on the intensity range of individual members (Titze and Maxfield, 2017), thereby making it potentially effective in deterring wild animals. We include this strategy under the predation deterrence hypothesis and suggest that beyond the deterrence of predators, it can be also used to describe the function of music to deter potentially dangerous animals in general, such as elephants, buffaloes, and gorillas. Although it is a part of the credible signaling hypothesis (which includes coalition signaling), we call our hypothesis predation deterrence to make a distinction between signaling among humans and signaling by humans toward non-human animals. According to our hypothesis, the strategy to repel any potential non-human threat is ornate and loud, especially when implemented in large numbers instead of being quiet and inconspicuous (Knight and Lewis, 2017).

While discussing the possible reasons for the origin of music, the occurrence of music in the context of a parent and infant cannot be ignored. The use of lullabies by the mother is shown to be a human universal and is the second most identifiable type of music after dance songs (Trehub, 2001; Mehr et al., 2019). A probable explanation for these observations is that music is a result of kin selection where music enhances the bonding between the mother and the infant. Fancourt and Perkins (2018) showed that infant-directed singing by mothers can induce more closeness compared to talking or playing. Furthermore, music is suggested to be a part of a unique well-coordinated multimodal exchange involving touch, eye contact, body movement, and infant-directed speech (Feldman, 2012; Pérez and Español, 2016; Dissanayake, 2021; Trehub, 2021; Hilton et al., 2022). This multimodal exchange could represent a primordial communicative system of information since prosodic changes and intonation of the mother toward the infant (infant-directed speech) along with touch and eye contact create a foundation for stable relationships later in life (Dissanayake, 2000; Koulomzin et al., 2002; Falk, 2004). Within this communication system, touch is specifically shown to play a crucial role in displaying parental attention. Specifically, affectionate touch by the parent which consists of gentle stroking and holding has been shown to play an important role in facilitating parent–infant bonds and social bonds along with impacting infant somatosensory development (Hertenstein, 2002; Bigelow and Williams, 2020; Carozza and Leong, 2021). The importance of the role of touch has also been studied in non-WEIRD societies such as the Aka, where mothers regularly carry and breastfeed their infants (Hewlett et al., 1998; Hewlett and Lamb, 2002; Konner, 2010, 2016). Importantly, Velandia et al. (2010) showed that the increase in vocal interactions between a mother and infant is dependent on skin-to-skin contact. However, to date, there is no evidence for a direct link between tactile cues and the propensity to sing to infants, especially in non-WEIRD societies. If music originated to signal attention (Mehr et al., 2021) instead of directly increasing social bonding between mother and infant, we propose that touch could be a prerequisite for singing behavior. In other words, the prevalence of touch could be crucial in initiating parental singing during parent–infant interactions. Therefore, in this study, we also investigate whether touch (through carrying) influences the likelihood of parental singing by studying the unique context of children being carried by the mother as well as alloparents in hunter-gatherer societies.

Here, we test the coalition signaling and predation deterrence hypotheses, both within the overarching credible signaling hypothesis. In addition, we study the proposed link between touch and singing in a parent–infant context. We conducted our study with a hunter-gatherer population: Mbendjele BaYaka (hereafter: “BaYaka”) in the Republic of Congo. The BaYaka are an egalitarian community with semi-nomadic (move from one seasonal camp to another every few months) lifestyles (Woodburn, 1982; Lewis, 2002; Salali and Migliano, 2015; Thompson, 2018). The BaYaka forage for wild food sources daily, such as fish, fruits, nuts, tubers, mushrooms, leaves, caterpillars, and meat, but they also engage in some crop cultivation and trade (Kitanishi, 1995; Lewis, 2002; Veen et al., 2023). Foraging activities are gendered among the BaYaka, where women generally focus on the gathering of fruits, nuts, and tubers while men preferably target meat, high-hanging fruits, and honey (Thompson, 2018; Veen et al., 2023). Music and dance are an integral part of the BaYaka society which dictates their day-to-day lives (Lewis, 2002, 2013; Knight and Lewis, 2017; Oloa-Biloa, 2017). The BaYaka exhibit music in both the individual and group levels, especially, BaYaka women, who often sing during foraging (Lewis, 2013) as well as in larger gatherings such as spirit plays (Oloa-Biloa, 2017). The BaYaka song structure consists of an ever-present gradient in terms of the complexity of the song. When they are alone in the forest, the song can either consist of a single note or display an alternation between the chest voice and to head voice, hence giving the impression of multiple singers instead of one (pers. observation by J.L). In addition, the BaYaka often showcase a complex polyphonic song structure intertwined with polyrhythms during social contexts such as spirit plays (*Mokondi Massana*) (Lewis, 2009; Knight and Lewis, 2017; Oloa-Biloa, 2017).

For our study, we observed the singing behaviors of BaYaka women during foraging activities in the forest, especially, when the women were searching and digging for wild tubers which are an essential part of the BaYaka diet (Salali, 2017; Veen et al., 2023). Previous studies on hunter-gatherers have largely focused on musical performances during stand-alone large-scale events such as rituals and ceremonies but rarely consider musical activities well-integrated with daily activities such as foraging contexts. Importantly, BaYaka women often sing while walking in the forest and during subsistence activities. Hence, studying their singing behaviors during foraging trips offers an ideal context to test the aforementioned hypotheses. The BaYaka women often travel long distances in the forest (median travel distance = 3.93 km on human-made trails and 0.42 km off-trail; see Jang 2019a for more information) for foraging trips, allowing us to examine the predation deterrence hypothesis as the prevalence of wild animals increases deep in the forest. Tracks of leopards and gorillas have been regularly spotted during the foraging trips with occasional narration or accounts of encounters with wild animals by the women (pers. observation by K.R.L.J). Moreover, the women are often accompanied by infants on foraging trips, which allows us to simultaneously study the effect of carrying an infant (a cue for touch) on singing behavior. Importantly, childcare in BaYaka societies is not restricted to mothers but extended to non-maternal caregivers, including genetically unrelated individuals (Meehan, 2009; Bogin et al., 2014; Meehan et al., 2014; Jang et al., 2022).

To test our hypotheses on the evolutionary function of singing, we used the following measures. The probability of singing was measured as the probability (yes or no), with which BaYaka women sang during a tuber searching and digging bout in the forest. First, we predicted that BaYaka women would sing more during foraging with an increase in the number of individuals in the foraging group, especially in the presence of less familiar individuals, who are socially less close to the focal women (coalition signaling hypothesis). When socially closer individuals are present in larger groups, we expect the individuals to use more efficient ways of communication such as talking as singing requires more effort (Hagen and Bryant, 2003; Mehr et al., 2021). By “more efficient”, we mean that language is an explicit and precise communication tool to immediately acquire information regarding resources (Lewis, 2013). In addition, we investigated the predation deterrence hypothesis. Based on prior observations, BaYaka women were reported to sing loudly in a synchronized choral manner, while walking in the forest which they reported would deter and prevent the startling of wild animals such as buffalos, elephants, gorillas, and leopards (Lewis, 2009; Knight and Lewis, 2017; Salali, 2017). As animal densities would be higher in the deep forest far away from villages and decrease in an area close to human settlements due to hunting pressure for bushmeat (Wilkie and Carpenter, 1999; Hennessey and Rogers, 2008; Tweh et al., 2015), we expected the women to be more vulnerable to the potential threat from wild animals once they moved further away from the village. Furthermore, the foraging women are thought to increase their singing frequencies when present deep in the forest and in larger numbers during menstruation. It is suggested that singing repels predators and animals that could be potentially attracted to the smell of the blood of these foraging women (Lewis, 2009, 2014). Therefore, being in large groups could enable them to incorporate loud synchronized singing behavior along with conspicuous body movements that could be influential in warding off potential non-human threats (Jordania, 2011, 2015; Lewis, 2014; Salali, 2017). In addition, singing loudly in large groups could reduce the risk of startling a distant potentially dangerous animal, such as elephants or gorillas, decreasing the risk that foragers would be attacked.

Hence, we predicted that BaYaka women would sing more especially in large groups when they were distant from the village (where the risk of encountering dangerous animals was presumed to be higher). Lastly, we also predicted that BaYaka women would sing more frequently the longer they carried infants in a foraging bout (see methods for more information), as a measure of touch.

## Methods

### Participants and data description

The data used for this study were collected in a BaYaka community near the village of *Djoube*, along the *Motaba* River in the *Likoula* department of the north-western Republic of the Congo by H.J. and K.R.L.J. The BaYaka community is often referred to as “Aka”, “Baaka”, “Baka”, and “Mbendjele” (Kitanishi, 1995; Bombjaková, 2018; Jang et al., 2019a). The data were collected from March to August 2015 and 2016 (Jang et al., 2019a,b) as part of a larger project investigating the navigation strategies of BaYaka women. During the 230 total observation days, H.J. and K.R.L.J. conducted continuous focal sampling of five focal women (estimated mean age = 31 years, range = 26–41; Supplementary Table 1 for a detailed summary) from the beginning (the moment the focal woman left the camp) to the end (when she returned to the camp) of the women foraging expeditions (Martin and Bateson, 2009). The same focal women were followed twice for consecutive days each in 2015 and 2016. In addition to foraging expeditions, data were also collected when the focal women traveled to nearby villages or went gardening for crop cultivation. Focal data included the behaviors, gestures, and vocalization of the focal woman, as well as her expedition group composition and whether she carried an infant (not necessarily her infant) at the point of observation. We recorded the focal women's diverse vocalizing actions using a voice recorder (Supplementary Table 4 for codes). We categorized these vocalizations based on the nature of communication, singing (roughly categorized into loud and soft), silence or non-vocalization, discussion (mentioning tuber species names or their locations), and conversation (talking about anything else other than food or the food locations). In this study, our main interest was the singing (both loud and soft) vocalization (Supplementary Figure 1 for the duration of all vocalizations). Additional metrics such as the species of the tuber collected (Supplementary Table 2) and Global Positioning System (GPS; Garmin-62)-based space-time stamps were accounted for in the dataset. See Supplementary Tables 3, 5 for full details of the data description.

### Definition and calculation of tuber searching and digging bout

The unit of our analyses is a tuber searching and digging bout, which consists of a starting point where a focal woman first exhibited tuber digging behavior and an end point where she exhibited a non-digging behavior before the next digging behavior (that would indicate the start of the next searching and digging bout). When the focal woman exhibited walking or inspection behaviors between two digging behaviors, we considered the duration of those activities also as a tuber searching duration. It is difficult to define a tuber patch by observation. Hence, to identify whether walking occurred between two different tuber patches or within the same tuber patch, we defined a tuber patch based on the BaYaka women's digging and traveling behavior and calculated the 75th percentile of the duration of all the tuber searching and digging bouts. If any of the bout durations exceeded the 75th percentile, we considered those as bouts that included focal women's movement between two different tuber patches. We then excluded those bouts from our analyses as we did not consider this type of long-distance movement as part of the foraging activity (Supplementary Figure 2). To calculate the duration of the tuber searching and digging bouts, we used a customized function along with *aggregate* and *merge* in RStudio (R Core Team, 2020).

### Calculation of relevant variables

The response variable in the model was singing probability. This was a binary response as an indication of whether the focal woman sang (1) or not (0) during a tuber searching and digging bout. As predictor variables, we included group size, dyadic association index, distance from the village, and duration of carrying a baby. The group size was determined by counting the number of individuals present along with the focal individual during each behavior carried out by the focal woman and averaged per tuber searching and digging bout.

The Dyadic Association Index (hereafter “*DAI”*) was calculated as a proxy for bond strength (or social closeness) and the relationship between the focal individual and all the other individuals. The other individuals observed along with the focal women consisted of individuals (both men and women) from the same camp (either genetically related or not and including children) and BaYaka individuals from other villages. The DAI is the proportion of time that two individuals were observed together during foraging activities out of the amount of time that either of them was observed foraging in total, representing how likely are two individuals to forage together (Nishida, 1968; Cairns and Schwager, 1987). For our study, we calculated a yearly DAI value for each dyad (the proportion of time two individuals spent together in a year). The value was calculated for two separate years, 2015 and 2016. The DAI ranged from 0 to 1, with 1 indicating that individuals were always observed together.


$$\begin{array}{l} \text{DAI}=\text{AB}/\left[\left(\text{A}+\text{B}\right)-\text{AB}\right] \end{array}$$

where A (Focal Individual) = the total time individual A was observed; B (Individual present in the foraging group) = the total time individual B was observed; AB = the time A and B were observed together.

To determine social closeness between focal women and other individuals in a foraging group during the observed tuber searching and digging bouts, we averaged the DAI values across all focal-partner dyads per tuber searching and digging bout and included the average values in the statistical model.

Using the GPS coordinates, the distances between the location at each observation point and the village were calculated (km) with a formula based on the spherical law of cosines (Supplementary Figure 1). The distances from the village were then further averaged for all locations where the women had been during a tuber searching and digging bout. We observed that children aged between 1 month and 4 years were carried while walking (Lancy and Grove, 2011; Salali et al., 2019). As a result, a total of 13 individuals (N_boys_=5, N_girls_=8) were identified as children who were carried, with a mean age of 1.546 in 2015 (refer to Supplementary Figure 3 for more information on how the ages were calculated). We calculated the total duration of carrying an infant per tuber searching and digging bout to use it as a cue for touch. We observed that the focal women often carried or spent time with their infants during foraging trips or with the infants of other women in the foraging group when they were not digging.

### Statistical analysis

The dataset used for statistical analyses consisted of a total of 1,704 tuber searching and digging bouts (with mean ± SD: 1,797.795 ± 6,823.564 s for the duration of the bouts) from five focal individuals with similar observation efforts per woman (Supplementary Table 1). The foraging parties existed mainly of groups of females. Only one male was present in the foraging group in 53 of the total 1,704 bouts (3.1% of the bouts) with a mean ratio of 0.003 for all bouts (number of males to the total number of individuals in a foraging group). We fitted a Generalized Linear Mixed Model (GLMM) with a Binomial error structure and logit link function (Baayen, 2008). The model simultaneously tested the coalition signaling and predation deterrence hypothesis. We used singing probability as a binary response variable (18.54% of the bouts, *N* = 316 included singing; see Supplementary Table 1 for a detailed summary). The model consisted of the interaction between group size and DAI, the interaction between group size and distance from the village, and the duration of carrying a baby (19.31% of the bouts included the carrying of a baby, *N* = 329; with mean ± SD: 493.508± 3,103.675 s for the baby carrying duration; Supplementary Table 1 for a detailed summary) as predictors (Table 1, Supplementary Figure 2 for the frequency distribution of model variables). We included the focal individual as a random effect, with the DAI, duration of carrying a baby, and interaction between group size and distance from the village as random slopes (Supplementary Table 7). The remaining random slopes were deemed to be unidentifiable and hence discarded. Including random slopes was done to keep the Type I error probability at the desired level of 0.05 (Schielzeth and Forstmeier, 2009; Barr et al., 2013). The correlations between the random slopes and intercepts were not included in the model. The distributions of the predictor and response variables were monitored accordingly before model construction (Supplementary Figure 2). All the predictors were then z-transformed to a mean of zero and standard deviation of one to ensure easier interpretation of model coefficients (Schielzeth, 2010). We used the *glmer* function of the package lme4 (Bates et al., 2015). The model converged and the full-null model comparison was carried out using a likelihood ratio test (Dobson, 2002) to avoid multiple testing where the null model consisted of the intercept and the random structure of the full model (Forstmeier and Schielzeth, 2011). By omitting the levels of the random effects one at a time, the model's stability was evaluated based on the calculated coefficients and standard deviations (Nieuwenhuis et al., 2012). The stability was estimated through customized scripts. Overall, the model was found to be of acceptable stability except for the intercept (Supplementary Figures 10, 13). Potential problems of collinear predictable variables were checked by analyzing the variance inflation factors (VIFs) using the *vif* function of the car package (Field 2005, Fox and Weisberg, 2019). None of the predictor variables were found to be collinear (Highest VIF = 1.3895 and 1.2064 for DAI and baby carrying duration, respectively). Furthermore, we used a parametric bootstrap to obtain confidence intervals of model estimates (function *bootMer* of the package lme4; *N* = 1,000 bootstraps). The confidence intervals were the widest for the intercept (C_95%_ = [−2.239, −1.205]) and DAI (C_95%_ = [−0.894, −0.121] (Supplementary Figures 8, 9). The explained variance was calculated using the *r.squaredGLMM* function of the MuMIn package (Barton, 2020). The marginal (only fixed effects) and conditional (fixed and random effects) R-squared values were found to be 0.2216 and 0.2954, respectively (Nakagawa et al., 2017). The graphs for the model were plotted in R (R Core Team, 2020) using the sjPlot (Lüdecke, 2020) and visreg packages (Breheny and Burchett, 2017; see Supplementary Figures 5, 6, 8 for alternative plots). All the analysis was done in R Studio (version 4.0.2, R Core Team, 2020). In addition to the main model, we ran a *post-hoc* model to test whether the focal women were more likely to sing specifically when they carried a baby for longer durations in bouts. To do so, we only used tuber and searching bouts where the baby was present in the foraging group.

**Table 1:** Model overview.

**Type**	**Hypothesis tested**	**Response**	**Predictors**	**Random effect**
GLMM (binomial)	Coalition signaling, predation	Singing probability	Group size^*^DAI, Group size^*^Distance from village, Duration of carrying baby	Focal ID

## Results

**Figure 1:**
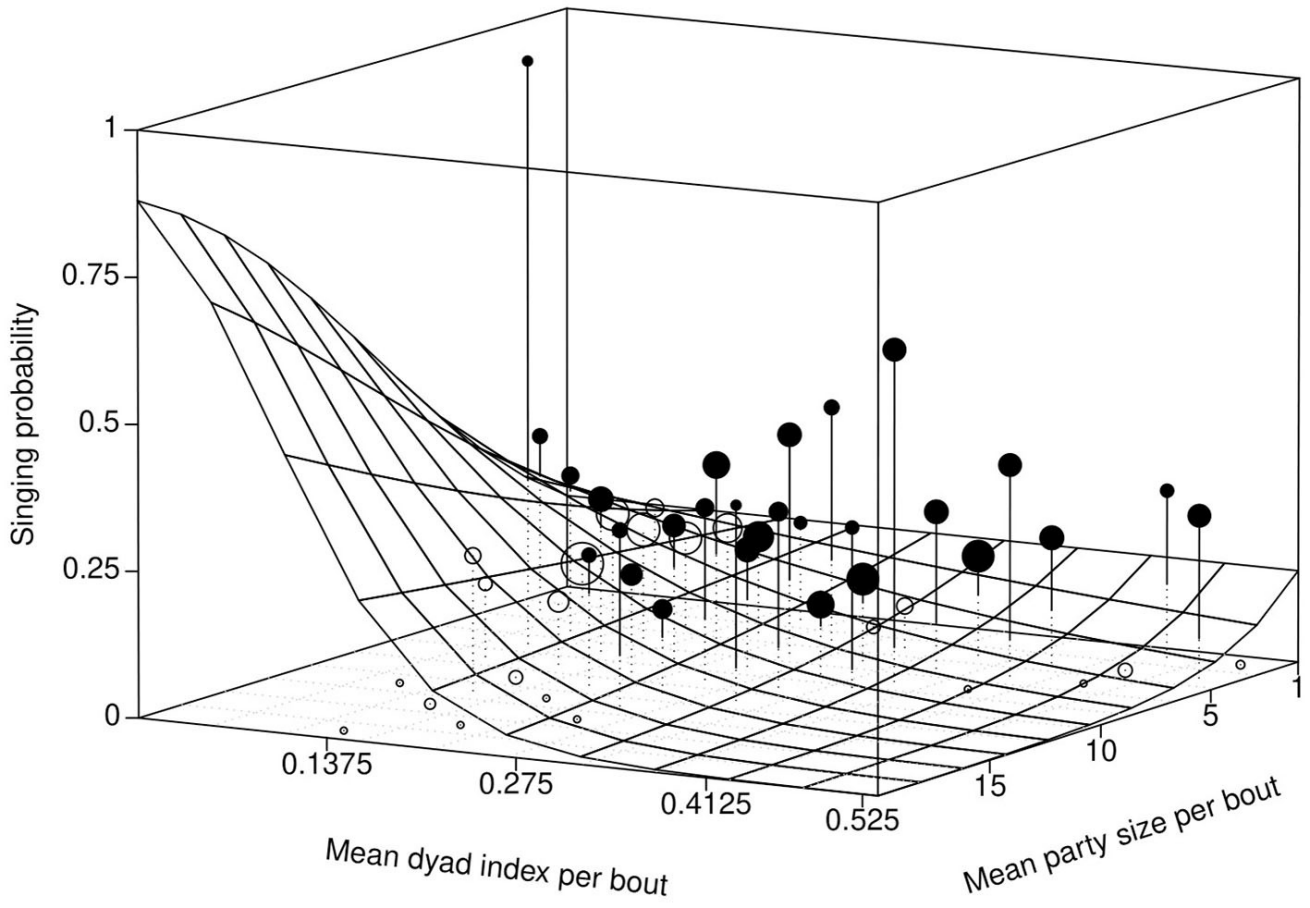
Effect of interaction between mean dyadic association index (DAI) and average party size on singing probability. The fitted model (surface; conditional on the distance from the village and duration of carrying a baby being at their average) and singing probability per level of the cell. The filled points indicate values above the fitted model while the open points depict values below the fitted model. The size or volume of the points indicates the number of individual data points per cell (Range = 1 to 244 points per cell. Mean=34.08 ± 44.7578). Mean group size has a strong positive effect on singing probability for low values of DAI, but the singing probability decreases steeply with an increase in the DAI for large party sizes. The focal women were more likely to sing more when present in large groups of less familiar individuals but were less likely to sing when present in large groups of familiar individuals (Estimate = −0.3782, SE = 0.1150, *p* = 0.0008). An alternative 2D version of this relationship (showing the effect of interaction between mean dyadic association index (DAI) and average party size on singing probability) is given in Supplementary Figures 3–7.

**Figure 2:**
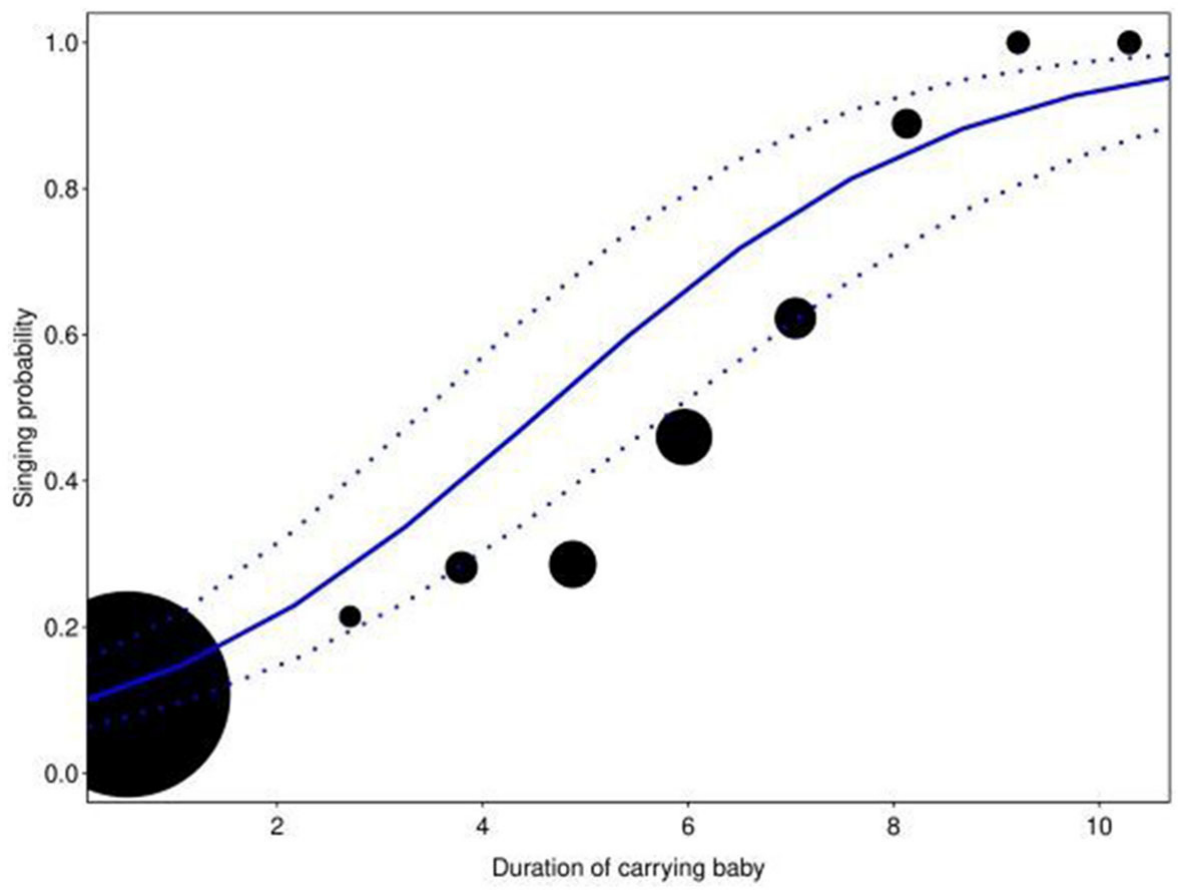
Effect of log-transformed duration of carrying baby (in s) on singing probability. The circles represent the amount of time (in s) the focal woman carried a baby. The size of the circles indicates the number of data points for particular ranges of duration (total N=14 to 1,375). The blue line represents the fitted model with the dashed lines representing the upper and lower confidence intervals, respectively. Singing probability increases with an increase in the duration of carrying a baby (Estimate = 1.2554, SE = 0.1295, *p* < 0.0001).

The full model showed significance when compared with the null model (full-null model comparison: χ^2^ = 30.7812, df = 6, *P* < 0.0001). In general, the singing probability of the women increased with a decrease in average DAI, with a more prominent increase when the women foraged in large groups compared to small ones. However, when in the presence of familiar individuals (high average DAI), the focal women were more likely to sing when they foraged in small groups compared to large groups (Estimate = −0.3782, SE = 0.1150, *P* = 0.0008; Table 2, Figure 1). In contrast, the interaction between group size and distance from village did not have a significant effect on the response (Estimate = −0.1272, SE = 0.0940, *P* = 0.1762), nor did the single term of distance from the village (after reducing the non-significant interaction). The duration of carrying a baby had a strong positive impact on singing probability within the foraging bouts (Estimate = 1.2554, SE = 0.1295, *P* < 0.0001; Table 2, Figure 2). In addition, the effect of duration of carrying a baby on singing probability remained strongly significant (Estimate = 1.4497, SE = 0.1694, *P* < 0.0001; Refer to Supplementary Tables 6, 7 for a detailed summary) in the *post-hoc* model (only consisting of bouts in which the baby was present in the foraging group). Therefore, the more time the focal woman under observation carried the baby herself (instead of having it carried by others), the higher the probability that she would sing.

**Table 2:** Results of the singing probability model (together with estimates, standard errors, significance tests, confidence intervals, as well as minimum and maximum of model estimates when excluding focal individuals one at a time to test model stability).

**Effect**	**Estimate**	**SE**	**χ2**	***P***	**95% C.I. (lower)**	**95% C.I. (upper)**	**Min**	**Max**
(Intercept)	−1.7014	0.2629	-	^(1)^	−2.239	−1.205	−1.908	−1.475
Group size^a^	−0.0178	0.0836	-	^(1)^	−0.185	0.141	−0.088	0.064
Dyadic association index (DAI)^b^	−0.5007	0.1914	-	^(1)^	−0.894	−0.121	−0.624	−0.399
Distance from village^c^	0.0630	0.0926	0.4584	0.4984	−0.138	0.237	−0.015	0.137
**Duration of carrying a baby in s** ^**d**^	**1.2554**	**0.1295**	**16.9117**	**< 0.0001**	**0.994**	**1.529**	**1.194**	**1.423**
**Group size: dyadic association index (DAI)**	**−0.3782**	**0.1150**	**11.2730**	**0.0008**	**−0.623**	**−0.164**	**−0.498**	**−0.346**

^(1)^Not indicated because of limited interpretability.

^a^Z-transformed; mean ± SD of the original values: 4.7899 ± 2.8756; Min = 1, Max = 20.

^b^Z-transformed; mean ± SD of the original values: 0.1934 ± 0.1276; Min = 0, Max = 0.5361.

^c^Z-transformed; mean ± SD of the original values: 4.9673 ± 1.6566; Min = 1.4778, Max = 7.8004.

^d^Z-transformed; mean ± SD of the original values: 493.5076 ± 3103.675; Min = 0, Max = 5094.

The bold values are the estimates/effects of the predictor variables on singing probability that are significant.

## Discussion

The singing probability model contrasted two key hypotheses on the origins of singing behavior, the coalition signaling and the predation hypotheses. Our results were consistent with the coalition signaling hypothesis as the focal women sang more frequently in larger groups of less familiar individuals and less frequently in larger groups consisting of more closely bonded individuals. In the presence of closely bonded individuals, singing probability decreased with an increase in group size. However, our results were not in line with the predation deterrence hypothesis.

Forming highly cooperative relationships with less familiar individuals between groups is an important component of human societies (Hagen and Bryant, 2003). Cooperation is important not only during the process of joint food acquisition, such as dam-fishing, nut-cracking, and tuber digging, but can also result in the shared knowledge of food locations and handling skills (Hagen and Bryant, 2003; Oloa-Biloa, 2017; Bombjaková, 2018; pers. observations by K.R.L.J and H.J). The effects of social connections with less familiar partners could, therefore, be beneficial over the long term as these members may possess skills and knowledge that closely bonded individuals may not have and can contribute to the labor needed for certain food-finding and handling techniques in future (Laden, 1992; Lewis, 2013). Bahuchet (1992) reported that several BaYaka groups unite for cooperative activities such as hunting and singing in large groups for festivals.

However, joint foraging can also result in conflicts if acquired food or information about food locations is not equally shared or is monopolized (pers. observation by H.J and K.R.L.J, Lewis, 2002; Oloa-Biloa, 2017). An increased proportion of less familiar and less related individuals during temporary foraging alliances will likely result in an increased probability of conflicts regarding equal sharing. In our study, although some of the individuals from the same camp or a different village are relatively less socially close with the focal women, they are united through crucial shared values such as egalitarianism, spreading positive emotions (such as “joy” or *bisengo*), and monogamy (Lewis, 2002, 2015, 2019; Knight and Lewis, 2017; Oloa-Biloa, 2017). Violations of these values can involve discussion and reinforcement of these values through songs and stories (Oloa-Biloa, 2017). Therefore, we also propose the possibility that song production in the BaYaka could have played an important role in minimizing the likelihood of potential conflict or mitigating existing conflicts during foraging. There are several possibilities for potential conflicts during foraging that have to be considered by the foraging women such as the locations on where to dig, who gets to use the iron tools which are essential for digging (pers. observation by K.R.L.J and H.J), and through stealing (Bombjaková, 2018, pers. observations by K.R.L.J and H.J). Through reinforcement of values such as spreading positive emotions within the continuously changing foraging alliances, cooperation and foraging success is expected to maintain and improve. Apart from singing, other strategies can be also incorporated to resolve conflicts during foraging such as avoidance (not foraging together with people with past disputes; pers. observation by K.R.L.J and J.L), announcing or warning other women publicly to not steal from the house gardens, hiding of tubers by placing them under leaves in baskets or on roofs of empty houses (theft occurred in 7 out of 1,704 bouts and hiding in 37 out of 1,704 bouts during our study; the percentage is 0.4% and 2.2%, respectively), and oral concealment of the location of a new tuber patch from the rest of the camp by foraging women (Bombjaková, 2018, pers. observation by K.R.L.J).

Our results are best explained by the coalition signaling hypothesis, supporting the idea that music could have originated as a tool to signal the intent to establish and strengthen temporary potentially conflict-free alliances with less familiar individuals, especially when they are large in number. In addition, song production could have been used as a mode to gain trust by acting as an “icebreaker” and assessing the intentions of less familiar members, thereby making music an inclusive activity that does not discriminate against individuals (Pearce et al., 2016). Among the BaYaka, the charisma and initiative of individuals are highly valued as such characteristics can positively contribute to cooperation and enhance trust among individuals (Oloa-Biloa, 2017). We suspect that song production also allows the focal women to gauge these valued characteristics in less familiar individuals.

Large groups usually act as an incentive to fuel more elaborate musical behavior with body movements. The BaYaka use music in a variety of different contexts (Lewis, 2013). One of the main occurrences of elaborate music behavior is during spirit plays (also known as *Mokondi Massana*). Spirit plays are mostly conducted in villages that also house a large number of genetically unrelated or less socially close individuals (Hill et al., 2011; Lewis, 2013, 2015). During these plays, there is no hierarchy dictating the rules of music performance regarding what can or cannot be included. However, the vocal contribution of each individual is still dependent on the melodies of other group members. In addition, the underlying principles dictating the musical principles of BaYaka could be also transferred to certain daily activities such as hunting (Widdess, 2012; Lewis, 2013). Personal observations by K.R.L.J have indicated that certain singing events also occur outside the context of spirit plays such as when the BaYaka go out or return from tuber foraging trips. These events could involve elaborate body movements such as stomping, clapping, and swaying during foraging expeditions. Among adults, practicing for big musical events seems quite universal in the ethnographic record (Merriam, 1964). Therefore, there is a strong possibility that singing during foraging excursions acts as rehearsals for large gatherings and events (Oloa-Biloa, 2017).

Furthermore, we suspect that singing during foraging excursions could enable the focal individuals to identify the less familiar individuals in a foraging group and assess their unique musical approaches or contributions. The less familiar individuals could introduce new melodies which can make the forest “happy” and bring joy to the focal individuals (Lewis, 2015). In BaYaka society, new melodies are often encouraged and highly valued. Hence, our findings are also in line with the role of music acting as an “icebreaker” among individuals (Pearce et al., 2016). In contrast, the reasons for the decrease in singing probability with increasing group size for high DAI values could be that singing invariably requires more effort and control over the voice than talking (Natke et al., 2003). Therefore, a group of related or familiar individuals could incorporate less costly vocalizations such as talking. We suspect that it could be easier for the focal women to use language to instruct familiar instead of less familiar individuals for the procurement of immediate knowledge regarding resources (e.g., where a tuber patch is). Language is more precise and explicit in facilitating communication of short-term intentions, goals, and knowledge sharing (Lewis, 2013). Since the focal women are socially close to these individuals already, trust could have already been achieved. Furthermore, we suspect the foraging women who are more familiar with each other will be less motivated to sing as they already know the melodies the socially close individuals could sing. Alternatively, we are open to the possibility of music being used to communicate with large groups of less familiar individuals to keep the group coordinated, as the group spread is expected to be larger, akin to the instances of “drumming” and call combination in chimpanzees and bonobos (Schamberg et al., 2016; Eleuteri et al., 2022; Fitzgerald et al., 2022). Music and dance could be an indicator of coalition quality, and stable coalitions tend to have more elaborate, complex, well-choreographed musical performances and are more synchronized (Hagen and Bryant, 2003; Mehr et al., 2021). However, our study unfortunately did not directly measure coalition quality which is a variable that determines how stable a coalition is or how collectively a group of people act to perform a task (Hagen and Bryant, 2003; Mehr et al., 2021).

In addition, we continued testing the credible signaling hypothesis by investigating the role of music in the protection against predators (Hagen, 2022). To test our predation deterrence hypothesis, the interaction between group size and distance from the village was selected as the proxy for predation risk. We predicted that singing probability would increase with an increase in distance from the village, especially when the women foraged in large groups. However, we found limited support for the predation deterrence hypothesis, as the interaction between group size and distance from the village had no significant effect on singing probability. A potential reason for the observed result is that the density of animals was probably not high in the areas where the women foraged for tubers, and hence singing may not have been necessary for these parts of the forest. Although tracks of animals such as leopards and gorillas were encountered along with repeated discussions in the camp regarding animal sightings, the density of these animals has not been regularly monitored (through camera traps) or verified in the area. Another possibility is that the animals were scared of humans in the area due to intense hunting pressure (Wilkie and Carpenter, 1999; Tweh et al., 2015). Due to the bushmeat trade, the animal density near the camp has decreased (pers. observation by K.R.L.J and M. Dzabatou). However, Knight and Lewis (2017) report instances of continuous, loud, and all-night singing by BaYaka women in the proximity of a predator. Therefore, future testing of the predation deterrence hypothesis should incorporate analyses of the singing behavior of women who reside in camps that are deeper in the forest—more than 15 km from villages—where there is sufficient evidence of the presence of predators or dangerous animals (through sightings, camera footage, and vocalizations).

In parallel, the total duration of carrying a baby had a significant positive effect on singing probability. The results were in line with our predictions and showed a possible link between singing and tactile cues which could have an impact on parent–infant bonds (Pérez and Español, 2016; Dissanayake, 2021). Furthermore, the results from the *post-hoc* model indicated that the focal women were more likely to sing when they carried a baby compared to when the baby was in the foraging party, but was carried or just accompanied by alloparents. Among the BaYaka, the presence of alloparents in foraging trips affect mother's foraging productivity (Jang et al., 2022). Our results further suggest that the presence of infants and alloparents during mothers' foraging activities affect also mothers' singing behaviors during foraging and that touch plays a vital supplementary role in parent-infant singing behavior. BaYaka women yodel loudly or rhythmically pat their infants' backs whenever they start crying profusely to coax and console the unsettled baby (Lewis, 2013). Lewis (2013) suggested that this behavior could be critical in developing musical skills and important tools to learn to create music. Furthermore, children are encouraged by adults to sing at an early age, especially during singing events, and are frequently exposed to sounds produced by musical instruments such as drums (Lewis, 2002, 2013; Oloa-Biloa, 2017). Therefore, our results were expected and unsurprising. As yet, we did not directly assess whether the singing was associated with the demeanor of the infants (i.e., relaxed or not) which could be measured in future studies by expanding on the previous work done by Hewlett et al. (1998). Importantly, our result can act as a primer to carry out future studies where it is tested whether an interaction between touch and parental singing affects affiliative behavioral cues such as happiness or laughing behavior in the infant. In other words, we can investigate if touch in tandem with singing increases bonding and trust between the parent and infant by regulating the mood and emotions of the infant. Maternal body movements during touch have been shown to drastically reduce restlessness and crying behavior in infants (Esposito et al., 2013). We further suggest that such reduction of restlessness and keeping the infant quiet could prevent potential predation, especially from leopards, which are an important cause of adult and especially infant casualties in foragers (Athreya et al., 2011; Janmaat et al., 2014; Kandel et al., 2023). Future studies that compare our data to those collected in areas with higher predation risks will therefore be crucial to disentangle the variables that explain singing behavior in BaYaka women who carry babies. We stress the urgency of conducting studies in areas with high predation risk as wildlife is on an alarming decline due to the increasing bushmeat trade (Hennessey and Rogers, 2008; Van Vliet et al., 2019). Lastly, we suspect that singing could be a way to increase endorphins to lessen the burden of carrying a baby in a similar way to the use of sea shanties sung by sailors during hard labor tasks such as rope hauling (Milne, 2017). This could be investigated by considering an interaction between carrying a baby and energy expenditure in future studies.

In conclusion, our study sheds light on the possibility that music could have originated to signal coalition, particularly cooperative intent or intention to form coalitions within and between groups. In addition, music could have a potential impact on parent–infant bonds by working in tandem with parental touch with a hunter-gatherer community as the sole focus. However, the signaling role of music is likely dependent on the societal structure as not all communities exhibit communal or collective music-making (Rudge, 2019; Patel and Von Rueden, 2021; e.g., the Tsimane of Bolivia and Batek of Malaysia). According to Moser et al. (2021), the diversity in musical forms is potentially an outcome of the complex and variable nature of human social structures and cultural niches. Future research assessing coalition signaling in different societies should account for the role of music in signaling their particular structure or identity, and hence, its contribution to the preservation or cohesion of that community.

## Data availability statement

The data analyzed in this study is subject to the following licenses/restrictions: personal information regarding the identities of the individuals. Requests to access these datasets should be directed to chirag.chittar@gmail.com.

## Ethics statement

The studies involving humans were approved by Comité d'Ethique de la Recherche en Sciences de la Santé (N°095/MRSIT/IRSA/CERSSA). The studies were conducted in accordance with the local legislation and institutional requirements. Written informed consent for participation in this study was provided by the participants' legal guardians/next of kin. Written informed consent was obtained from the individual(s) for the publication of any potentially identifiable images or data included in this article.

## Author contributions

CC and KJ: conceptualization and writing—original draft preparation. HJ and KJ: data collection. CC, LS, KJ, and EL: formal analysis and investigation. CC, HJ, HH, JL, LS, EL, and KJ: writing—reviewing and editing. KJ: funding acquisition. All authors contributed to the article and approved the submitted version.
