# Supplementary material for: Music production and its role in coalition signaling during foraging contexts in a hunter-gatherer society
Source: Front Psychol. 2023 Nov 1;14:1218394. doi: 10.3389/fpsyg.2023.1218394 (PMC10646562; doi:10.3389/fpsyg.2023.1218394)
Supplement: Supplementary file 1 [file Table_1.docx]

***Supplementary Material***

**S.1. Equation to calculate distance. Used to calculate distance from camp and distance from village variables.**

**d=acos(sin φ_1_ ⋅ sin φ_2_ + cos φ_1_ ⋅ cos φ_2_ ⋅ cos Δλ ) ⋅ R**

where, φ_1_ is the latitude of the village; φ_2_ is the latitude of the current location; Δλ=the difference between the longitude of the current location and the village location and R=the mean radius of the earth which is 6371 kms. The angles were calculated in radians.

**S.2. Extraction of presence and absence of baby for the post hoc model**

A binary variable indicating whether a baby was present (yes) or absent (no) in the foraging group during a tuber searching and digging bout. The variable was first extracted for every day when the focal individual was followed and then for all the tuber searching and digging bouts (a baby was present in the foraging group in 908 bouts or 53.29% of the bouts and absent in 796 or 46.71% of the bouts). A total of 13 individuals (N_boys_=5, N_girls_=8) were identified as infants (individuals having ages between 1 month and 4 years were considered as infants; Lancy and Grove 2011, Salali et al 2019) with a mean age of 1.546 in 2015 (age range=0.1-4 years). Since there is an absence of birth records maintained by the BaYaka, the ages were estimated based on the birth orders provided by the families. Furthermore, the ages of known BaYaka individuals or Bantu were taken as anchor points. The anchor points were used as reference and the ages of the rest of the BaYaka individuals were estimated based on inter-birth intervals of 2.5 years (Hill and Hurtado 2017, Jang et al 2019a). The age estimation was done by H.J. and K.R.L.J.

# **TABLES**

**Table S.1. Table summary of distribution of key variables among the five focal women during the course of the study.**

|  | Woman 1 | Woman 2 | Woman 3 | Woman 4 | Woman 5 |
| --- | --- | --- | --- | --- | --- |
| Estimated age (at the start of the study in 2015) | 36 | 40 | 26 | 24.5 | 24.5 |
| Number of observations/bouts | 642 | 328 | 305 | 260 | 169 |
| Number of singing observations/bouts where the focal woman sang (%) | 91 (14.17%) | 28 (8.54%) | 109 (35.74%) | 51 (19.62%) | 37 (21.89%) |
| Average duration of singing (sec) | 48.9019 | 57.4908 | 137.7246 | 144.3269 | 198.0828 |
| Total duration of singing (%) | 31395 (3.74%) | 18857 (3.11%) | 42006 (8.77%) | 37525 (5.80%) | 33476 (6.80%) |
| Number of observations/bouts where a baby was present (%) | 316 (49.22%) | 168 (51.22%) | 159 (52.13%) | 147 (56.54%) | 118 (69.82%) |
| Number of observations/bouts where the focal woman carried a baby (%) | 54 (8.41%) | 33 (10.06%) | 141 (46.23%) | 42 (16.15%) | 59 (34.91%) |
| Total duration the focal woman carried a baby (%) | 148645 (17.73%) | 42705 (7.04%) | 165277 (34.49%) | 236806 (36.61%) | 247504 (50.25%) |

| **Row number** | **Vernacular name** | **Species code** | **Species** | **Family** | **Habitat** | |
| --- | --- | --- | --- | --- | --- | --- |
| 1 | Bendje | Unidentified | Unidentified | Unidentified | Garden |  |
| 2 | Bobaka | Manesc | *Manihot esculenta* | Euphorbiaceae | Forest |  |
| 3 | Bodiaka | Unidentified | Unidentified | Unidentified | Garden |  |
| 4 | Boma | Manesc | *Manihot esculenta* | Euphorbiaceae | Garden |  |
| 5 | Boyeba | Manesc | *Manihot esculenta.* | Euphorbiaceae | Forest |  |
| 6 | Ekule | Diosem | *Dioscorea semperflorens* | Dioscoreaceae | Forest |  |
| 7 | Epangi | Unidentified | Unidentified | Unidentified | Forest |  |
| 8 | Essouma | Diosch | *Dioscorea schimperana* | Dioscoreaceae | Forest |  |
| 9 | Ewala | Unidentified | Unidentified | Unidentified | Forest |  |
| 10 | Gomagoma | Unidentified | Unidentified | Unidentified | Forest |  |
| 11 | Ika | Colesc | *Colocasia esculenta* | Araceae | Garden |  |
| 12 | Jabangka | Unidentified | Unidentified | Unidentified | Forest |  |
| 13 | Kobo | Unidentified | Unidentified | Unidentified | Garden |  |
| 14 | Makeesoso | Manesc | *Manihot esculenta* | Euphorbiaceae | Forest |  |
| 15 | Mandiki | Unidentified | Unidentified | Unidentified | Forest |  |
| 16 | Mela | Diocum | *Dioscoreophyllum cumminsii* | Menispermaceae | Forest |  |
| 17 | Momosa/Mole | Unidentified | Unidentified | Unidentified | Forest |  |
| 18 | Mongemba | Afrlep | *Afrostyrax lepidophyllus* | Huacaeae | Forest |  |
| 19 | Ngangi | Diobur | *Dioscorea burkilliana* | Dioscoreaceae | Forest |  |
| 20 | Yoko | Unidentified | Unidentified | Unidentified | Garden |  |

**Table S.2. Table showing list of tuber species.**

**Table S.3. Table showing how data was recorded.** Each row representing an independent observation, with the focal name column displaying the name of the individual observed at a particular time. Vocalization column indicating the vocalization performed by the focal individual. The carry baby column highlighting whether the individual carrying a baby at the point of observation. Individuals in group showing the names of individuals present along with the focal individual at that point in time. Food type indicating the food type that was foraged. The behavior column indicating the behavior performed by the focal individual at that point in time supplemented by the behavior duration column showing the duration of the behavior (in sec).

| **Entire row number** | **Focal name** | **Vocalization** | **Carry baby** | **Individuals in group** | **Food Type** | | **Behavior** | **Behavior duration** |
| --- | --- | --- | --- | --- | --- | --- | --- | --- |
| **1** | Bote | Di | Nobaby | buma,bote,apel,dasa | | R | dig | 38 |
| **2** | Bote | Di | Nobaby | buma,bote,apel,dasa,anic | |  | s_ig | 59 |
| **3** | Bote | Si | Nobaby | buma,bote,apel,dasa,anic | |  | dep | 22 |
| **4** | Bote | Di | Nobaby | buma,bote,apel,dasa,roma,anic | | R | dig | 31 |
| **5** | Bote | Di | Nobaby | buma,bote,apel,dasa, | |  | cn | 31 |
| **6** | Bote | Nv | Nobaby | buma,bote,apel,dasa, | |  | dep | 14 |
| **7** | Bote | Si | Nobaby | buma,bote,apel,dasa, | |  | scu | 18 |
| **8** | Bote | Sa | Nobaby | buma,bote,apel,dasa,anic | |  | sca | 77 |

**Table S.4. Table showing various codes occurring in the dataset with suitable explanations.**

| **Behavior code** | **Behavior** | **Vocalization code** | **Vocalization** | **Food type code** | **Food Type** | |  |  |
| --- | --- | --- | --- | --- | --- | --- | --- | --- |
| S | Standing | Di | Discussion | R | | Tuber |  |  |
| Dep | Locomotion | Si | Loud singing |  | |  |  |  |
| A | Sitting | Nv | Non-vocalization |  | |  |  |  |
| Dig | Digging tubers | Sa | Soft singing |  | |  |  |  |
| Cn | Scanning | Co | Conversation |  | |  |  |  |
| Scu | Scanning up |  |  |  | |  |  |  |
| Sca | Scanning sitting |  |  |  | |  |  |  |
|  |  |  |  |  | |  |  |  |

**Table S.5. Explanations of important column names in the data for the singing probability model.**

| Column name | Explanation |
| --- | --- |
| bout_number | Bout number |
| food_type | Food type |
| food_code | Code for the concerned food type |
| bout_dur | Duration of bout |
| baby_dur | Duration of carrying a baby per tuber searching and digging bout |
| baby_pres_bout | Presence of a baby per tuber searching and digging bout |
| loud_dur | Duration of loud singing per tuber searching and digging bout |
| mean_secs_sm | Average seconds passed since midnight/time of the day |
| tot_number_food_bout | Total number of food collected per bout |
| YR | Year |
| Focal_name | Name of the focal subject |
| mean_days_arrival_camp | Average days since arrival to camp for the first time |
| mean_party_size | Average group size per bout |
| mean_behav_dur | Average duration of behavior per bout |
| mean_distance_from_camp_kms | Average distance from camp per bout |
| mean_distance_from_village_kms | Average distance from village per bout |
| mean_dyad_index | Average DAI per bout |
| MO | Month |
| DY | Day |
| H | Hour |
| M | Minute |
| S | Seconds |
| mean_endur | Average foraging endurance |
| LAT | Latitude |
| LON | Longitude |

**Table S.6. Post Hoc Model: Effect of interaction between party size and DAI (not shown in table), party size and distance from village (not shown in table), and baby carrying duration on singing probability only in bouts when the baby was present in the foraging group.**

| Effect | Estimate | SE | χ2 | P | 95% C.I  (Lower) | 95% C.I  (Upper) | Min | Max |
| --- | --- | --- | --- | --- | --- | --- | --- | --- |
| (Intercept) | -1.3694 | 0.2523 | - | ^(1)^ | -1.866 | -0.966 | -1.590 | -1.250 |
| Group size ^(2)^ | -0.1890 | 0.1665 | 1.7363 | 0.1876 | -0.475 | 0.101 | -0.333 | -0.087 |
| Dyadic association index (DAI)^(3)^ | -0.4511 | 0.2836 | 1.8276 | 0.1764 | -0.948 | 0.076 | -0.746 | -0.231 |
| Distance from village ^(4)^ | -0.0897 | 0.1544 | 0.4142 | 0.5199 | -0.367 | 0.182 | -0.190 | 0.052 |
| Duration of carrying a baby in s ^(5)^ | 1.4497 | 0.1694 | 17.3558 | <0.0001 | 1.202 | 1.738 | 1.360 | 1.685 |

^(1)^ not indicated because of limited interpretability

^(2)^  Z-transformed; mean ± SD of the original values: 6.0341 ± 2.7586; Min=2, Max=20

^(3)^ Z-transformed; mean ± SD of the original values: 0.2791 ± 0.1044; Min=0, Max=0.5361

^(4)^ Z-transformed; mean ± SD of the original values: 5.2835 ± 1.3902; Min=1.9280, Max=7.8004

^(5)^ Z-transformed; mean ± SD of the original values: 926.1421± 4205.425; Min=0, Max=50935

**Table S.7. Random slopes of all the models.**

| Random effect for all models | Model -Singing probability model (full) | Model-Singing probability (reduced) | Post-hoc Model |
| --- | --- | --- | --- |
| Focal individual | Intercept  DAI  Duration of carrying baby  Interaction between group size and distance from village | Intercept  DAI  Duration of carrying baby | Intercept  DAI  Duration of carrying baby  Interaction between group size and DAI  Interaction between group size and distance from village |

# **FIGURES**

**Fig. S.1. The frequency distribution for log-transformed bout duration for tuber digging, with the black dotted line indicating the 75th percentile.**

**Fig. S.2. Frequency distribution of all the model variables:** (A) mean group size per tuber searching and digging bout, (B) mean dyadic association index per tuber searching and digging bout, (C) log-transformed mean duration of carrying baby per tuber searching and digging bout, (D) mean distance from village per tuber searching and digging bout.

**Fig. S.3. 3-D plot showing the effect of interaction between mean dyadic association index (DAI) and average party size on singing probability when theta=60^o^.**

**Fig. S.4. 3-D plot showing the effect of interaction between mean dyadic association index (DAI) and average party size on singing probability when theta=120^o^.**

**Fig. S.5. Plot showing the effect of interaction between mean group size and Dyadic association index on singing probability.** We classified the DAI into two broad categories based on 874 the minimum and maximum values i.e., Low DAI or weak social bond dyads and High DAI or strong 875 social bond dyads (DAI=0 and 0.5361 respectively; 0.1934 ± 0.1276). Singing probability increases 876 steeply with increasing group size for individuals with weak social bonds with the focal woman 877 (Estimate=-0.3782, SE=0.1150, p=0.0008). However, singing probability decreases with increasing 878 group size from small initial values when the foraging group comprised of individuals with stronger 879 social bonds. Importantly, the x-axis is z-transformed with a mean of 0 and standard deviation of 1 880 (Original range of mean group size= Min=1, Max=20; 4.7899 ± 2.8756).

**Fig. S.6. Plot showing the effect of interaction between mean party size and dyadic association index (DAI) on singing probability.** DAI is set at its mean and 1 standard deviation below and above its mean (0.1934 ± 0.1276). Singing probability increases gradually with increasing party size at -1 SD while decreases gradually from initial low values at mean and +1 SD (Estimate=-0.3782, SE=0.1150, p=0.0008). The x-axis is z-transformed with a mean of 0 and standard deviation of 1.

**Fig. S.7. Contour plot showing the effect of interaction between mean group size and mean dyadic association index (DAI) on singing probability.** Group size and DAI constitute the x and y-axis respectively whereas singing probability is represented by the colour gradient. The distance between adjacent contours indicates steepness where, small distance signifies a steep increase or decrease and large distance indicates minimal increase/decrease. Singing probability increases steeply with increasing group size for small values of DAI (colour change from black to purple; Estimate=-0.3782, SE=0.1150, p=0.0008). However, singing probability decreases slightly from initial low values with increasing group size for large values of DAI (colour change from grey to black). Both the x and y-axis are z-transformed with a mean of 0 and standard deviation of 1 (Original range of mean group size= Min=1, Max=20; 4.7899 ± 2.8756. Original range of mean DAI= Min=0, Max=0.5361; 0.1934 ± 0.1276).

**Fig. S.8. Dot and whisker plot indicating confidence intervals for the full singing probability model.** Plot indicating confidence intervals of all the predictor variables. Notably, the confidence intervals are too wide for intercept (C_95%_ =[-2.261,-1.209]) and DAI (C_95%_ =[-0.872,-0.120]).

**Fig. S.9. Dot and whisker plot indicating confidence intervals for the reduced singing probability model.** Plot indicating confidence intervals of all the predictor variables of the singing probability model. Notably, the confidence intervals are too wide for intercept (C_95%_ =[-2.239,-1.205]) and DAI (C_95%_ =[-0.894,-0.121]).

**Fig. S.10. Stability plot for the full singing probability model without the random effects.** Plot indicating minimum and maximum estimates of all the predictor variables and overall stability of the singing probability model.

**Fig. S.11. Stability plot for the random effects of the full singing probability model.**

**Fig. S.12. Stability plot for the reduced singing probability model without the random effects.** Plot indicating minimum and maximum estimates of all the predictor variables and overall stability of the singing probability model.

**Fig. S.13. Stability plot for the random effects of the reduced singing probability model.**
