# Supplementary figures and images for: Music production and its role in coalition signaling during foraging contexts in a hunter-gatherer society
Source: Front Psychol. 2023 Nov 1;14:1218394. doi: 10.3389/fpsyg.2023.1218394 (PMC10646562; doi:10.3389/fpsyg.2023.1218394)

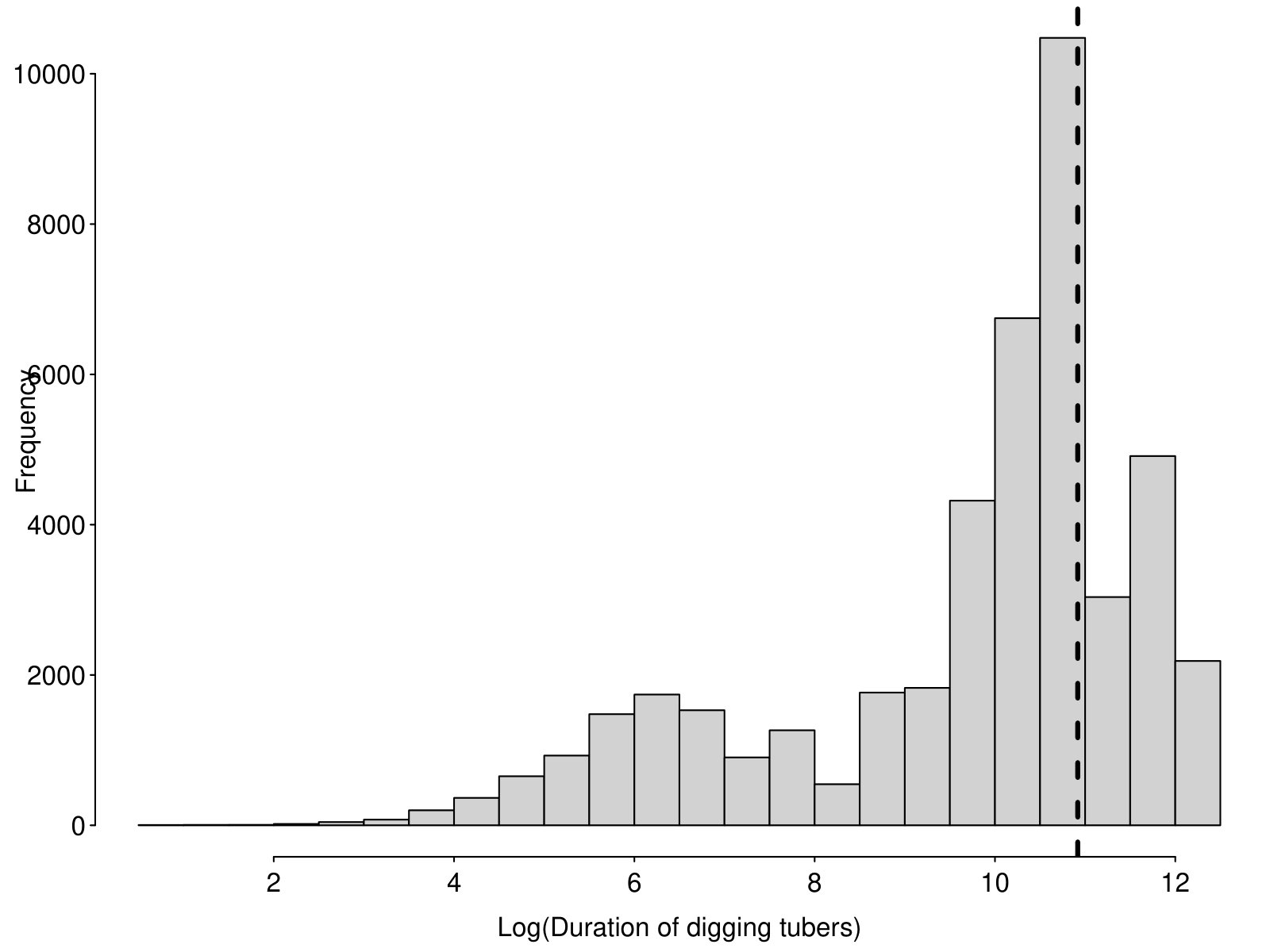

Supplement: Supplementary file 2 [file Data_Sheet_1.ZIP › Fig. S.1. The frequency distribution for log-transformed bout duration for tuber digging.jpeg]

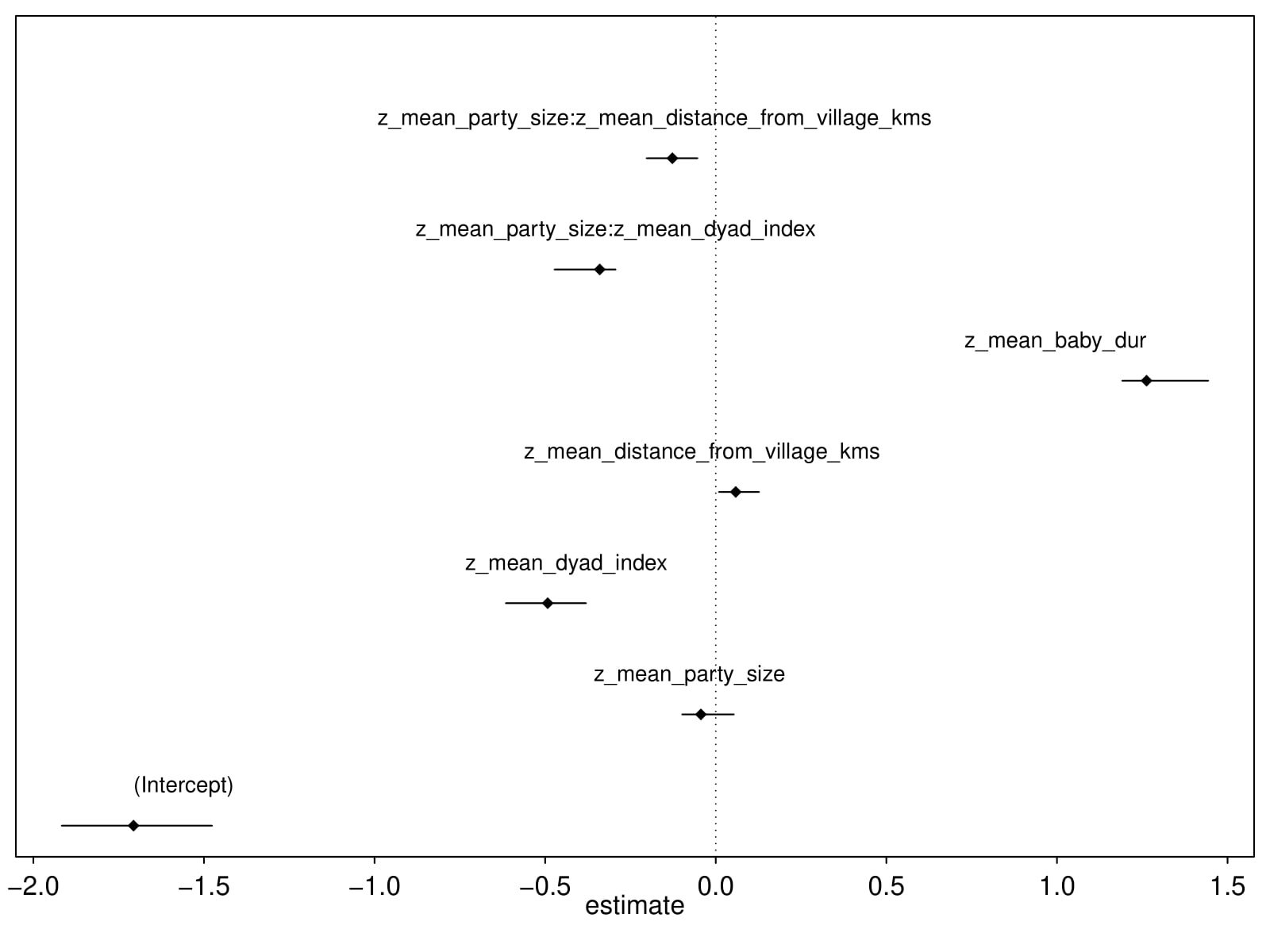

Supplement: Supplementary file 2 [file Data_Sheet_1.ZIP › Fig. S.10. Stability plot for the full singing probability model without the random effects.jpg]

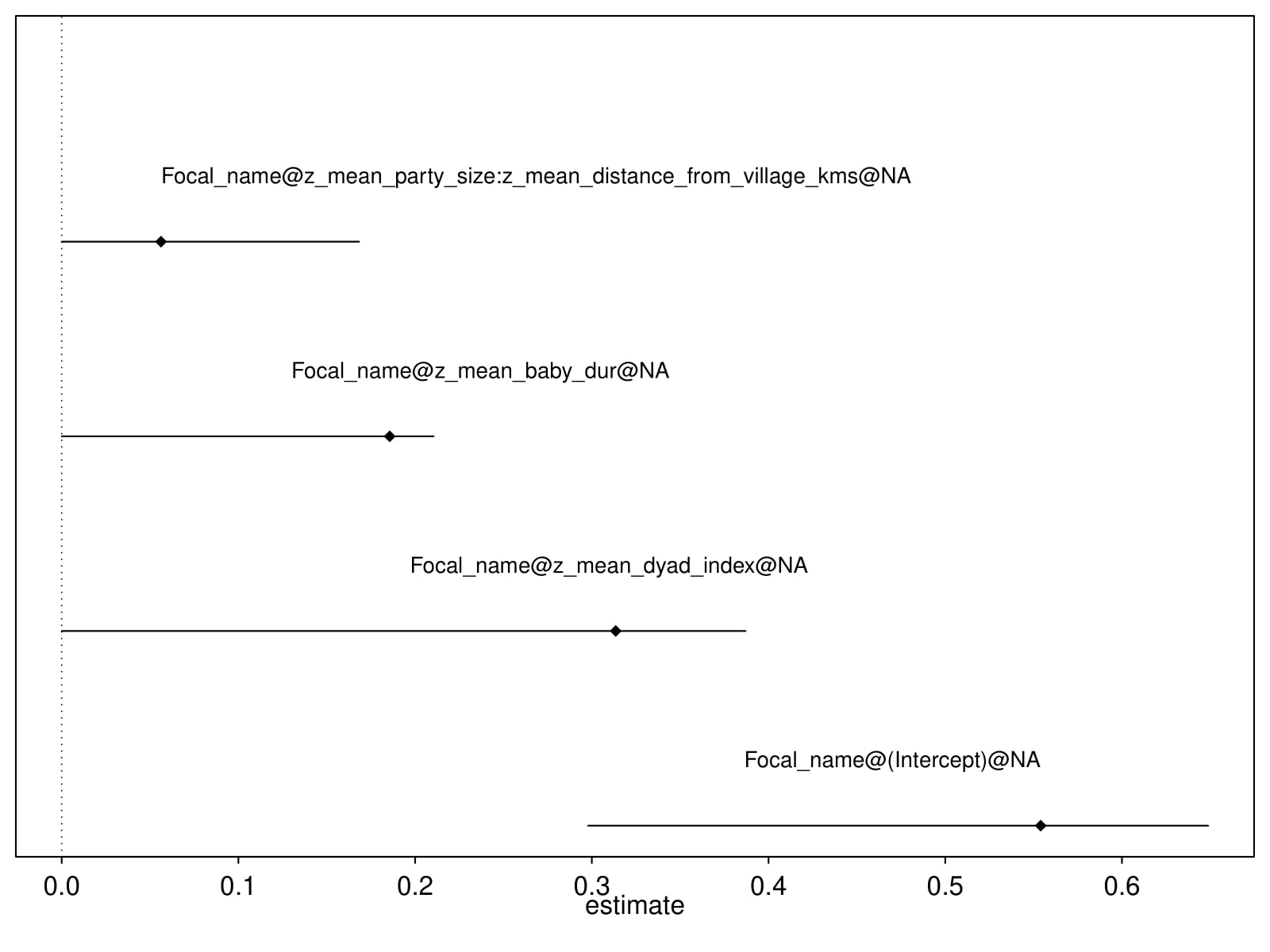

Supplement: Supplementary file 2 [file Data_Sheet_1.ZIP › Fig. S.11. Stability plot for the random effects of the full singing probability model..jpg]

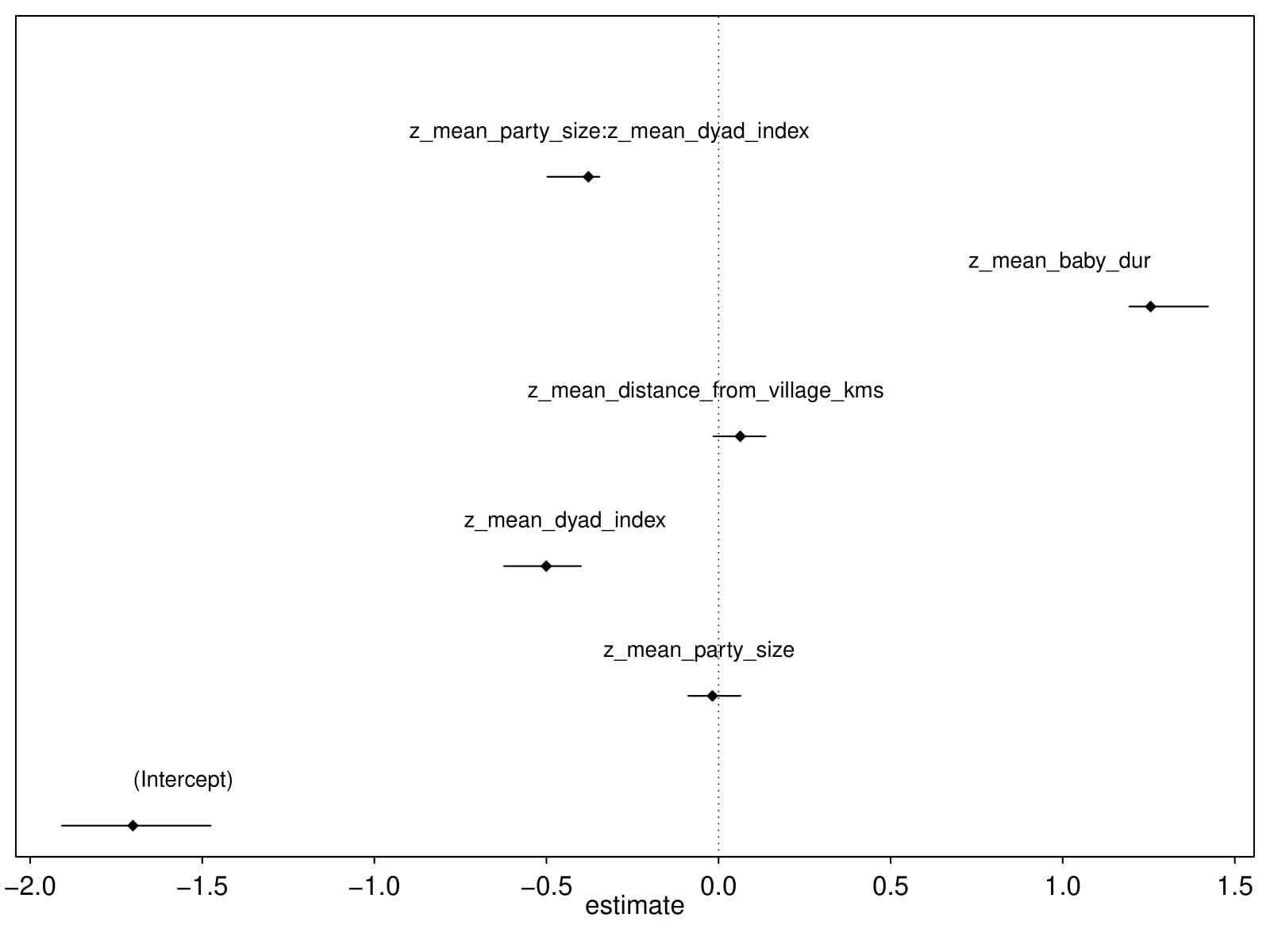

Supplement: Supplementary file 2 [file Data_Sheet_1.ZIP › Fig. S.12. Stability plot for the reduced singing probability model without the random effects..jpg]

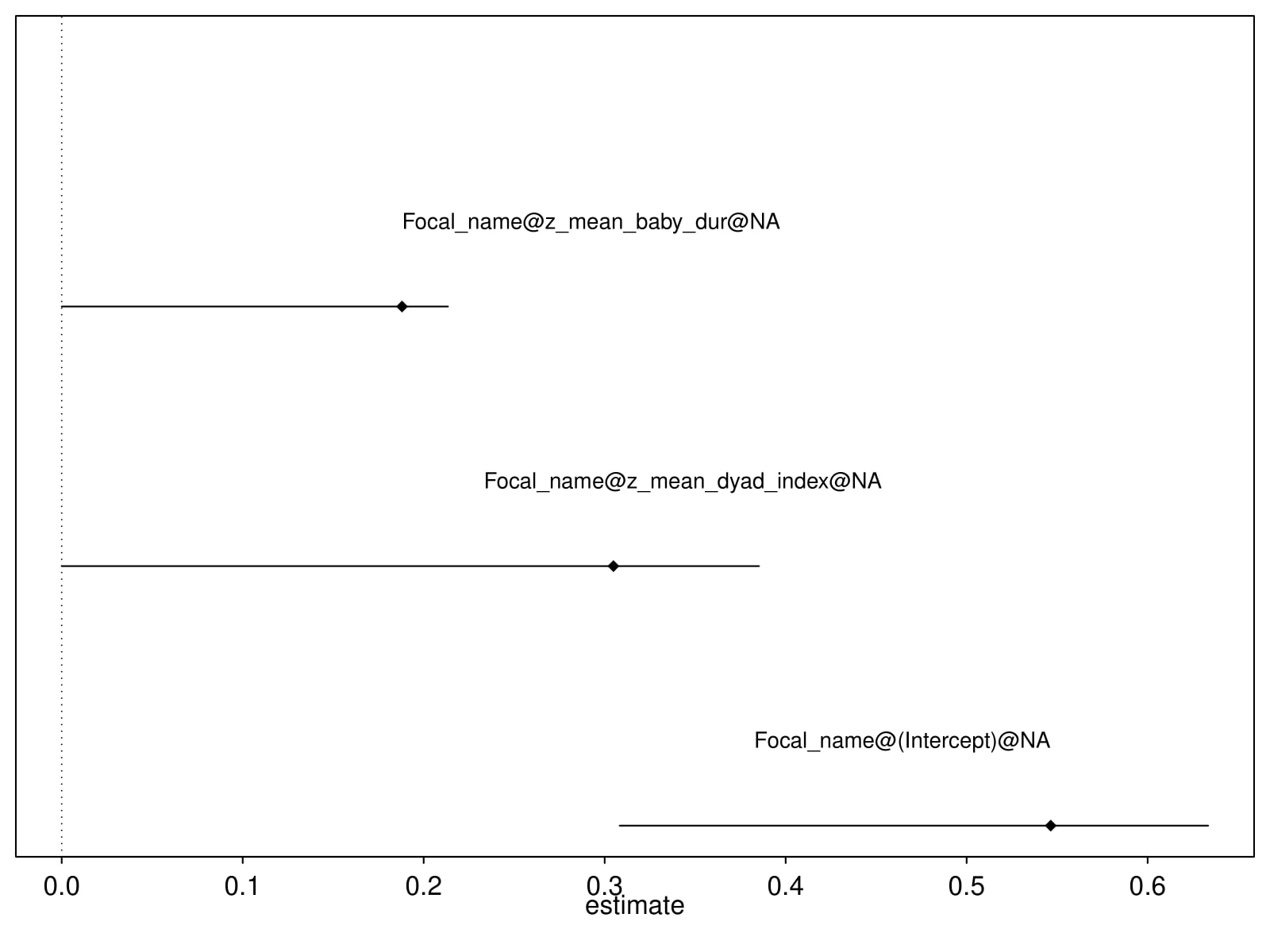

Supplement: Supplementary file 2 [file Data_Sheet_1.ZIP › Fig. S.13. Stability plot for the random effects of the reduced singing probability model..jpg]

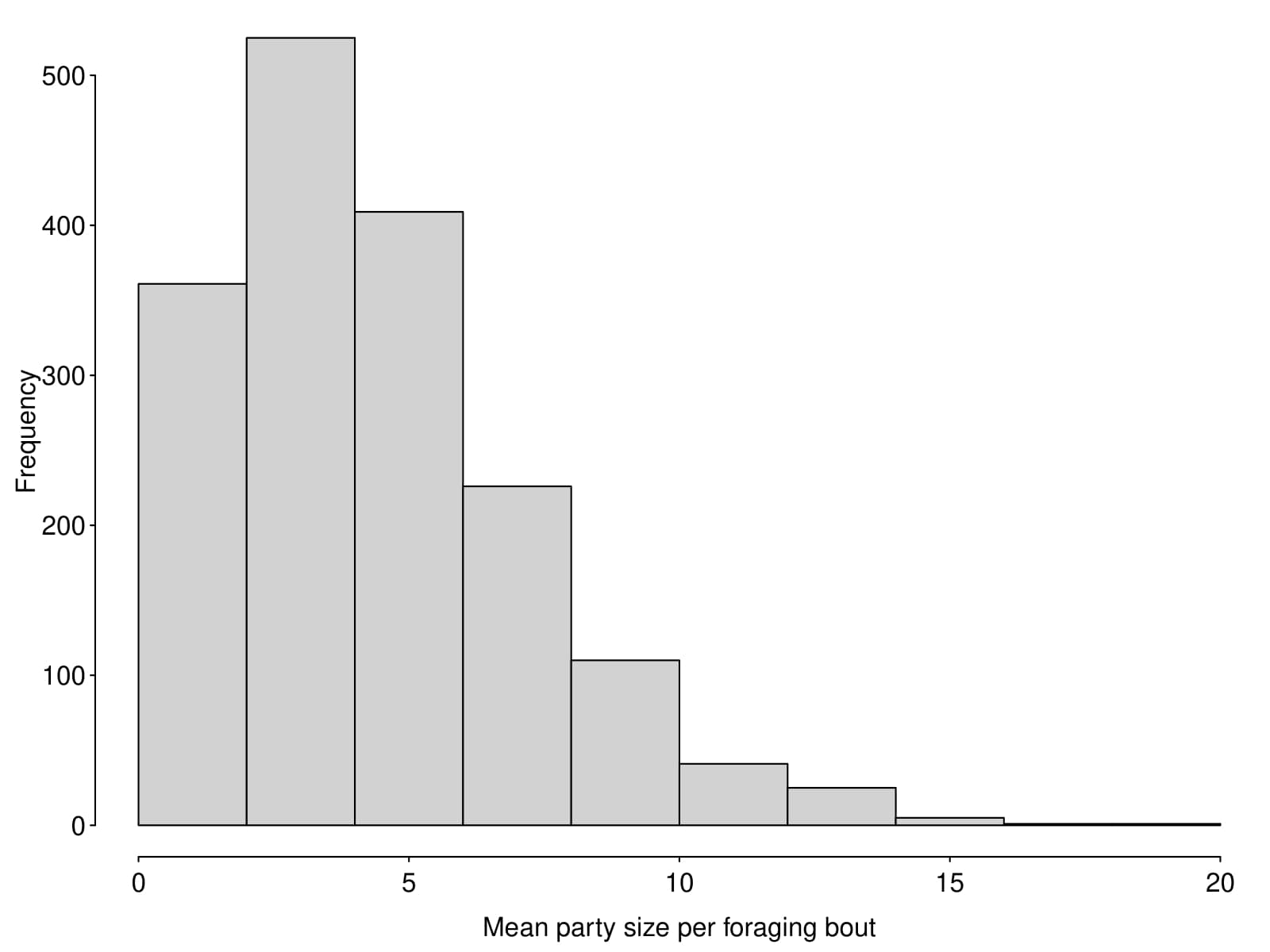

Supplement: Supplementary file 2 [file Data_Sheet_1.ZIP › Fig. S.2. Frequency distribution of all the model variables. (A) mean group size per tuber searching and digging bout.jpg]

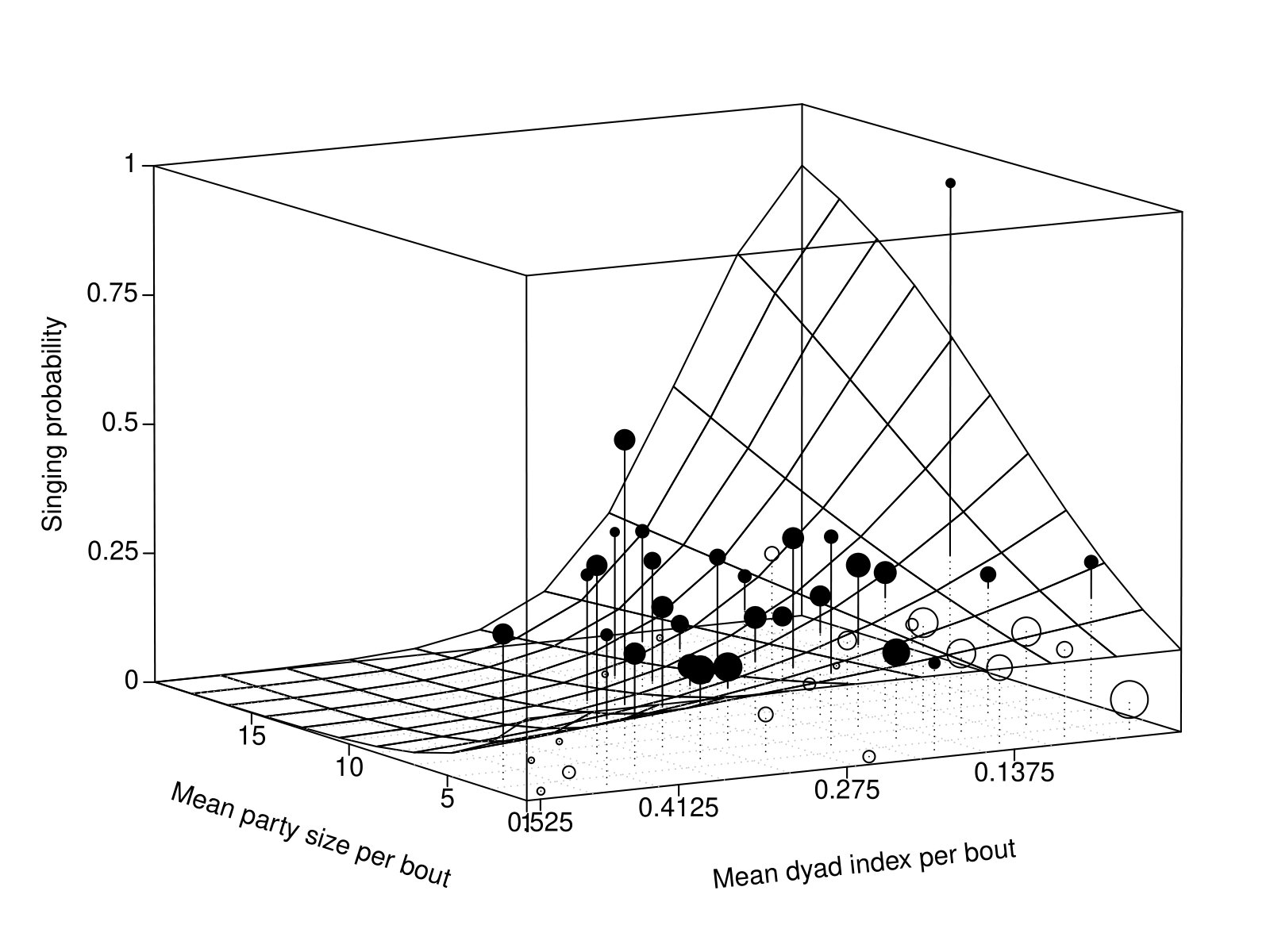

Supplement: Supplementary file 2 [file Data_Sheet_1.ZIP › Fig. S.3. 3-D plot showing the effect of interaction between mean dyadic association index (DAI) and average party size on singing probability.jpg]

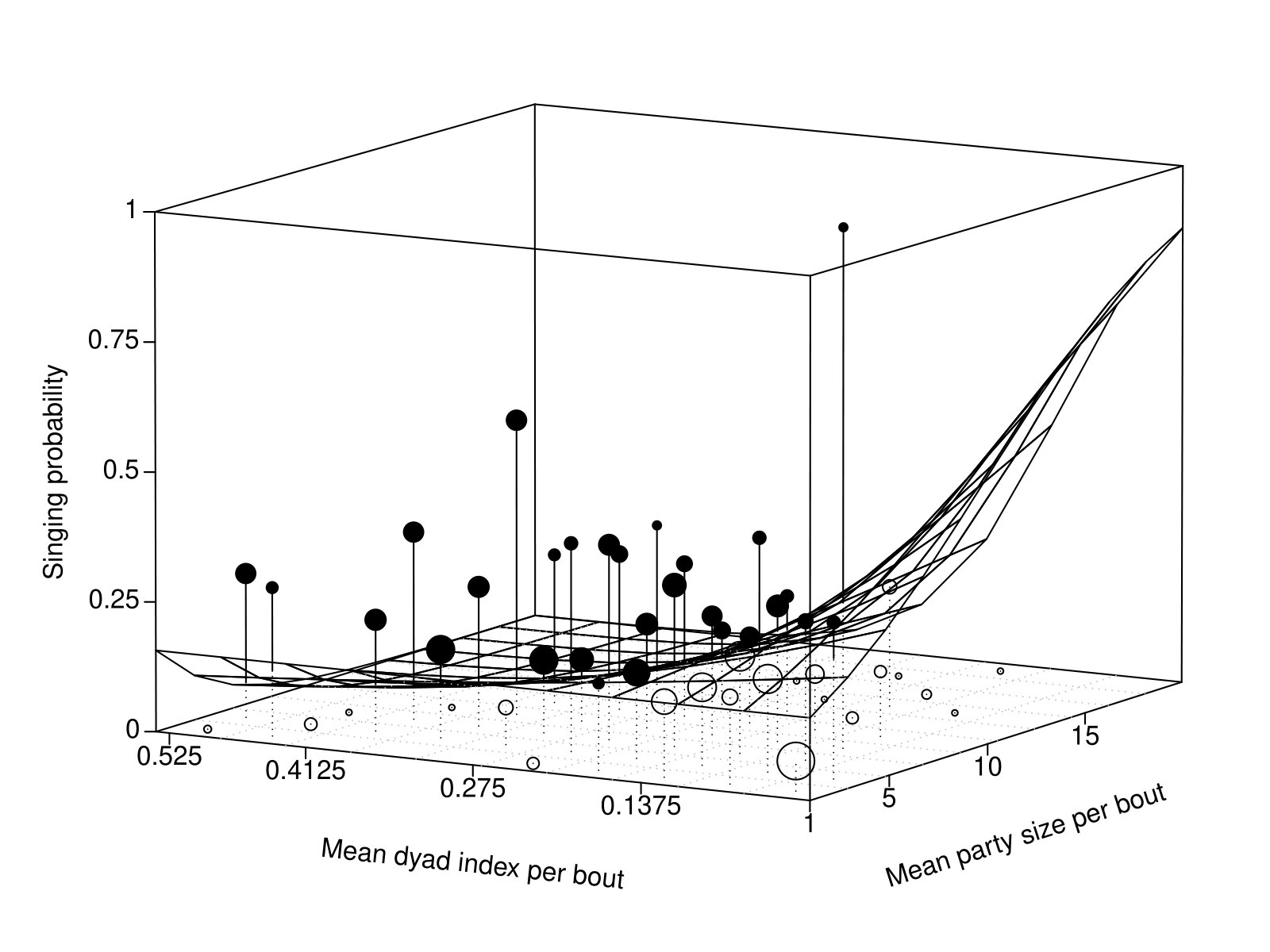

Supplement: Supplementary file 2 [file Data_Sheet_1.ZIP › Fig. S.4 3-D plot showing the effect of interaction between mean dyadic association index (DAI) and average party size on singing probability ..jpg]

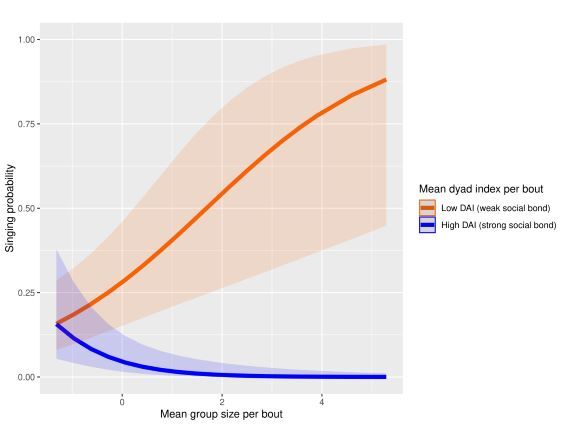

Supplement: Supplementary file 2 [file Data_Sheet_1.ZIP › Fig. S.5. Plot showing the effect of interaction between mean group size and the Dyadic association index on singing probability..jpg]

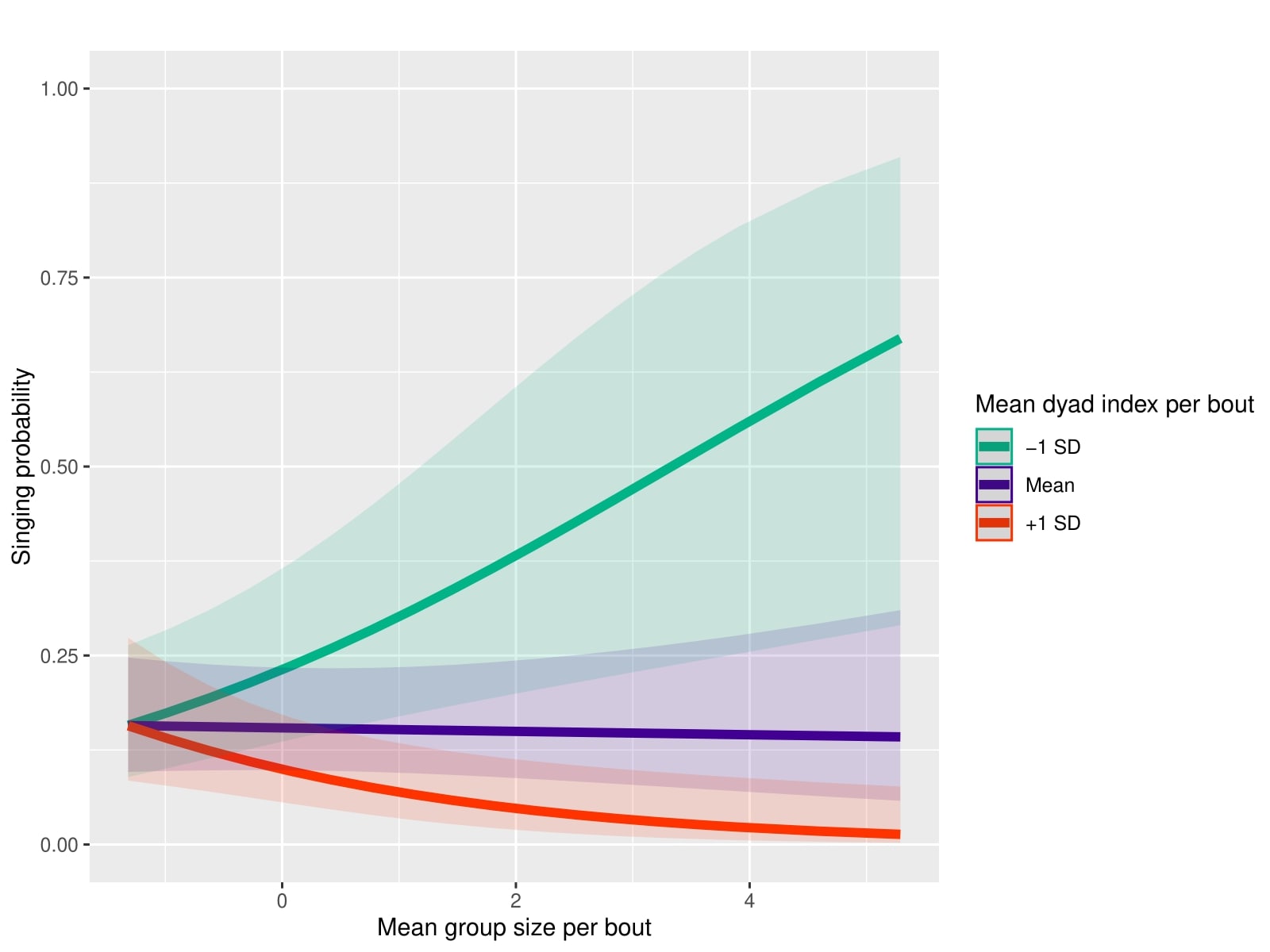

Supplement: Supplementary file 2 [file Data_Sheet_1.ZIP › Fig. S.6. Plot showing the effect of interaction between mean party size and dyadic association index (DAI) on singing probability..jpg]

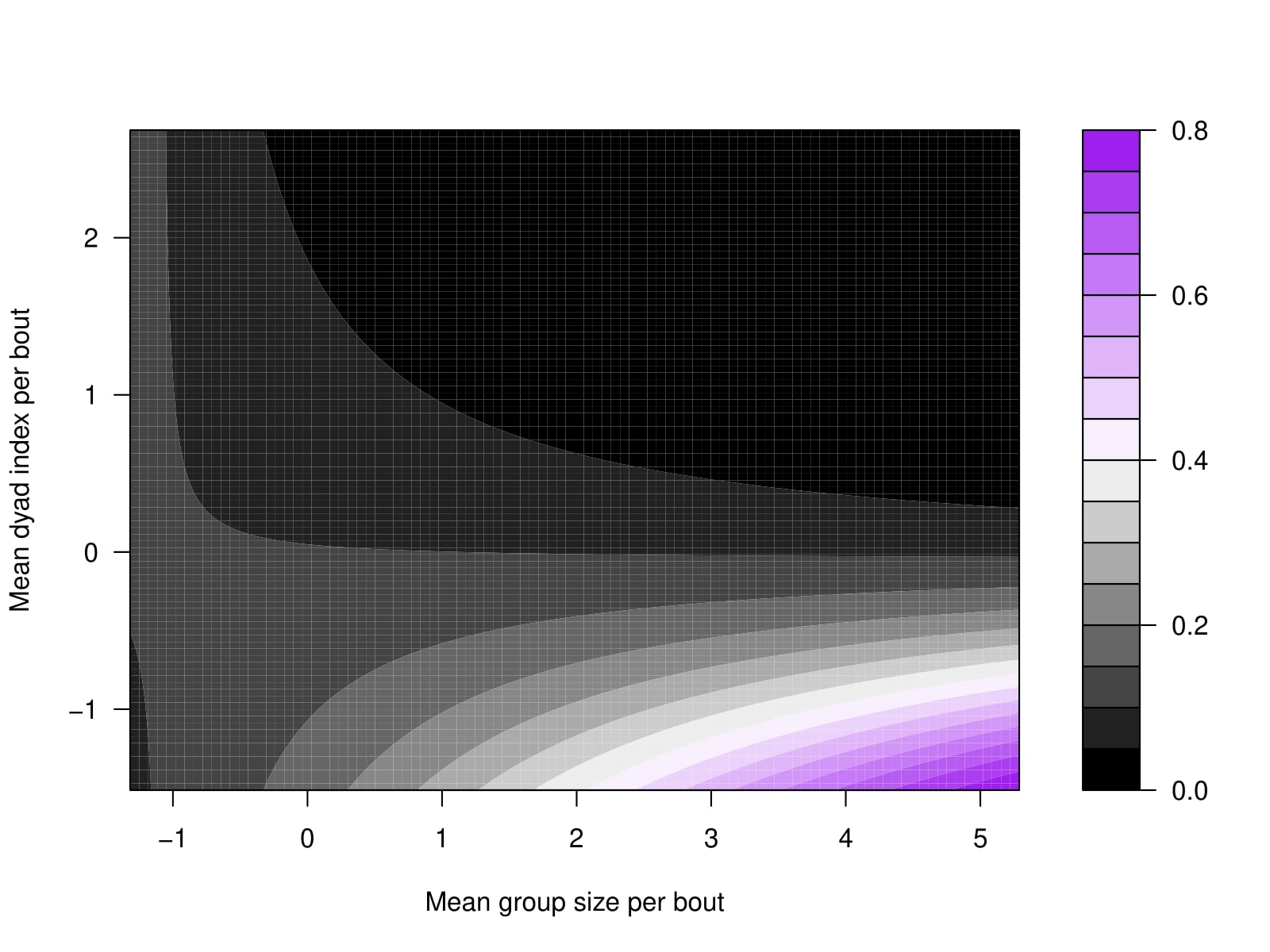

Supplement: Supplementary file 2 [file Data_Sheet_1.ZIP › Fig. S.7. Contour plot showing the effect of interaction between mean group size and mean dyadic association index (DAI) on singing probability..jpg]

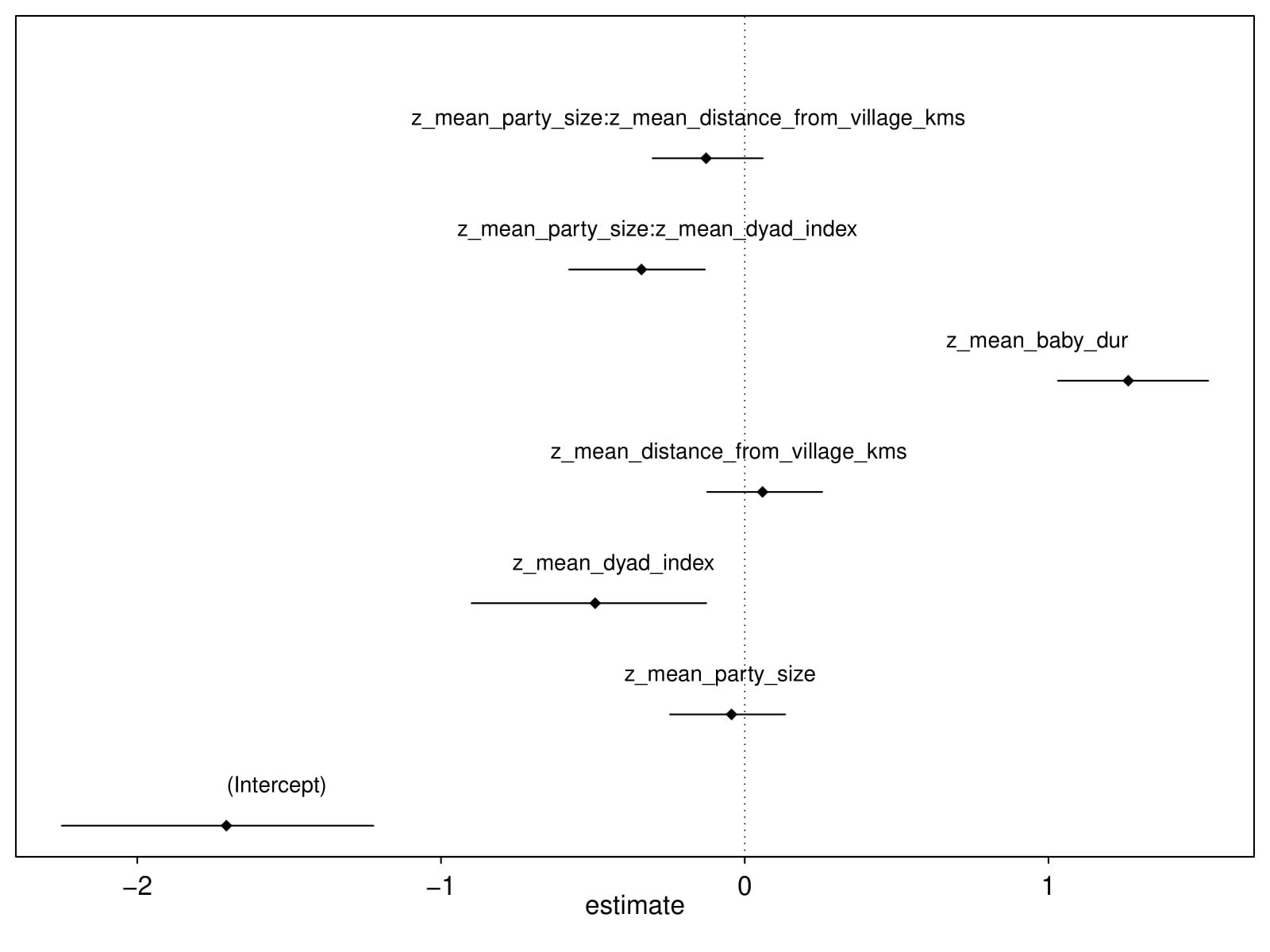

Supplement: Supplementary file 2 [file Data_Sheet_1.ZIP › Fig. S.8. Dot and whisker plot indicating confidence intervals for the full singing probability model..jpg]

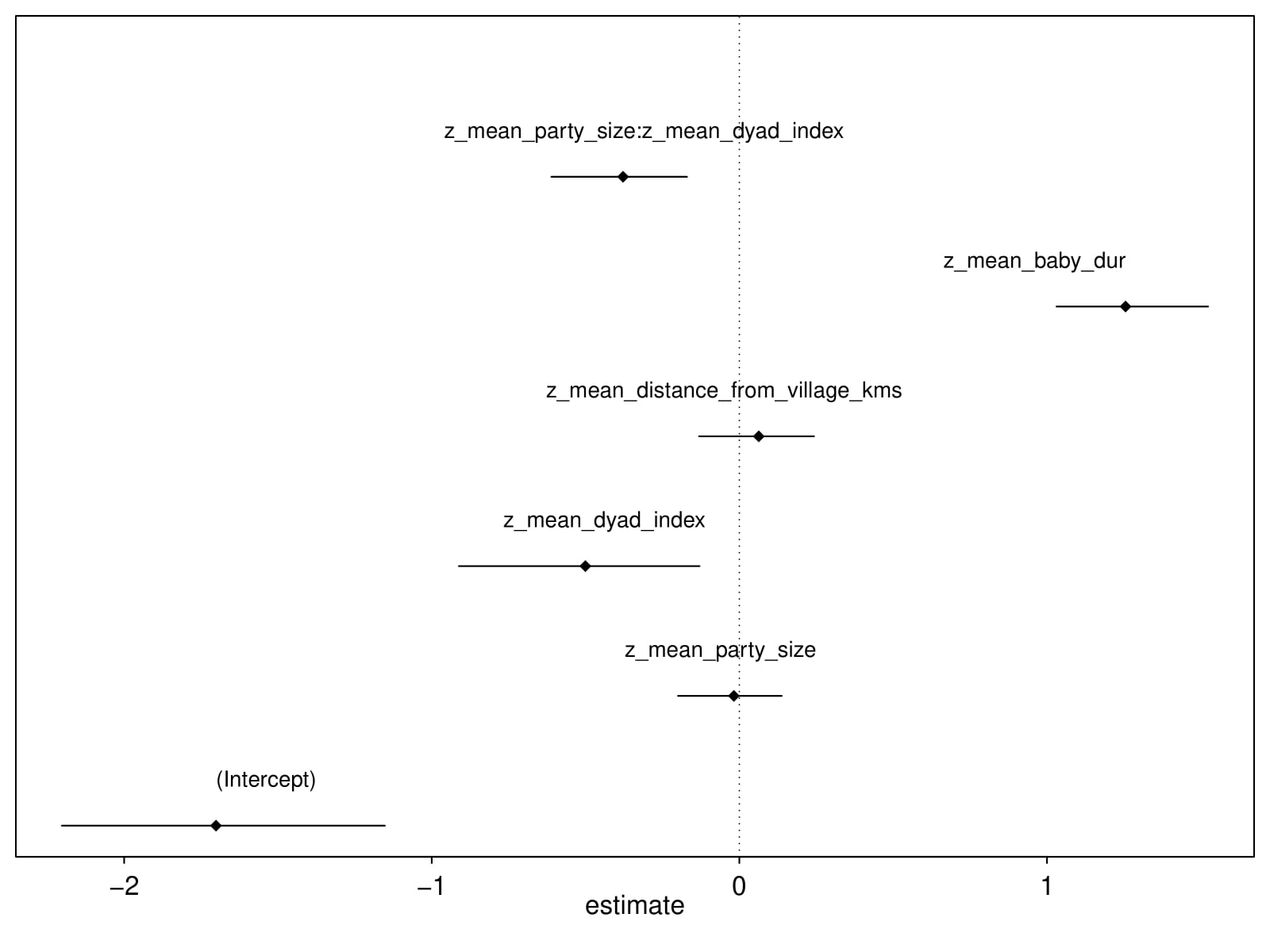

Supplement: Supplementary file 2 [file Data_Sheet_1.ZIP › Fig. S.9. Dot and whisker plot indicating confidence intervals for the reduced singing probability model..jpg]

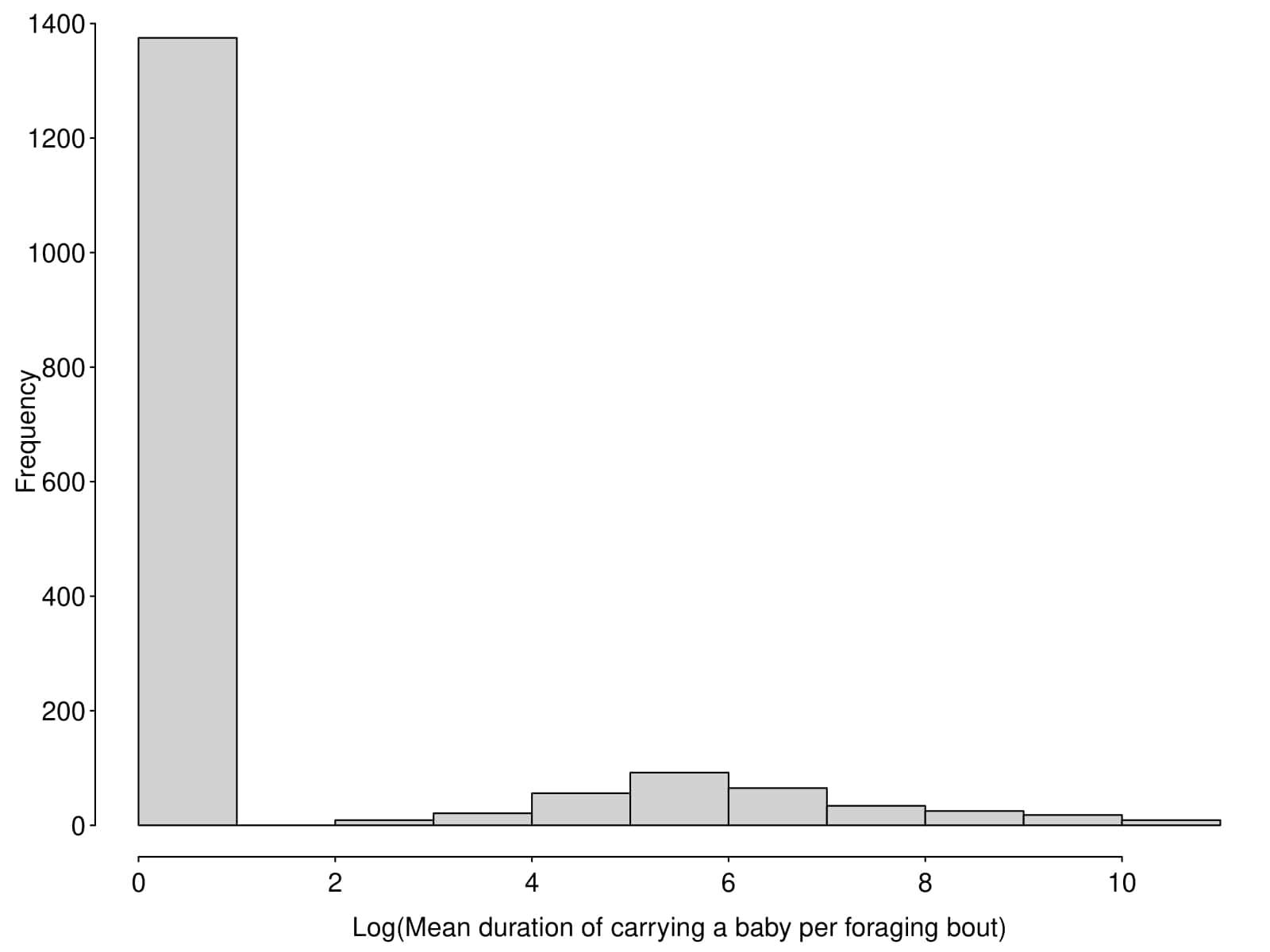

Supplement: Supplementary file 2 [file Data_Sheet_1.ZIP › Fig.S.2. Frequency distribution of all model variables. (C) log-transformed mean duration of carrying baby per tuber searching and digging bout.jpg]

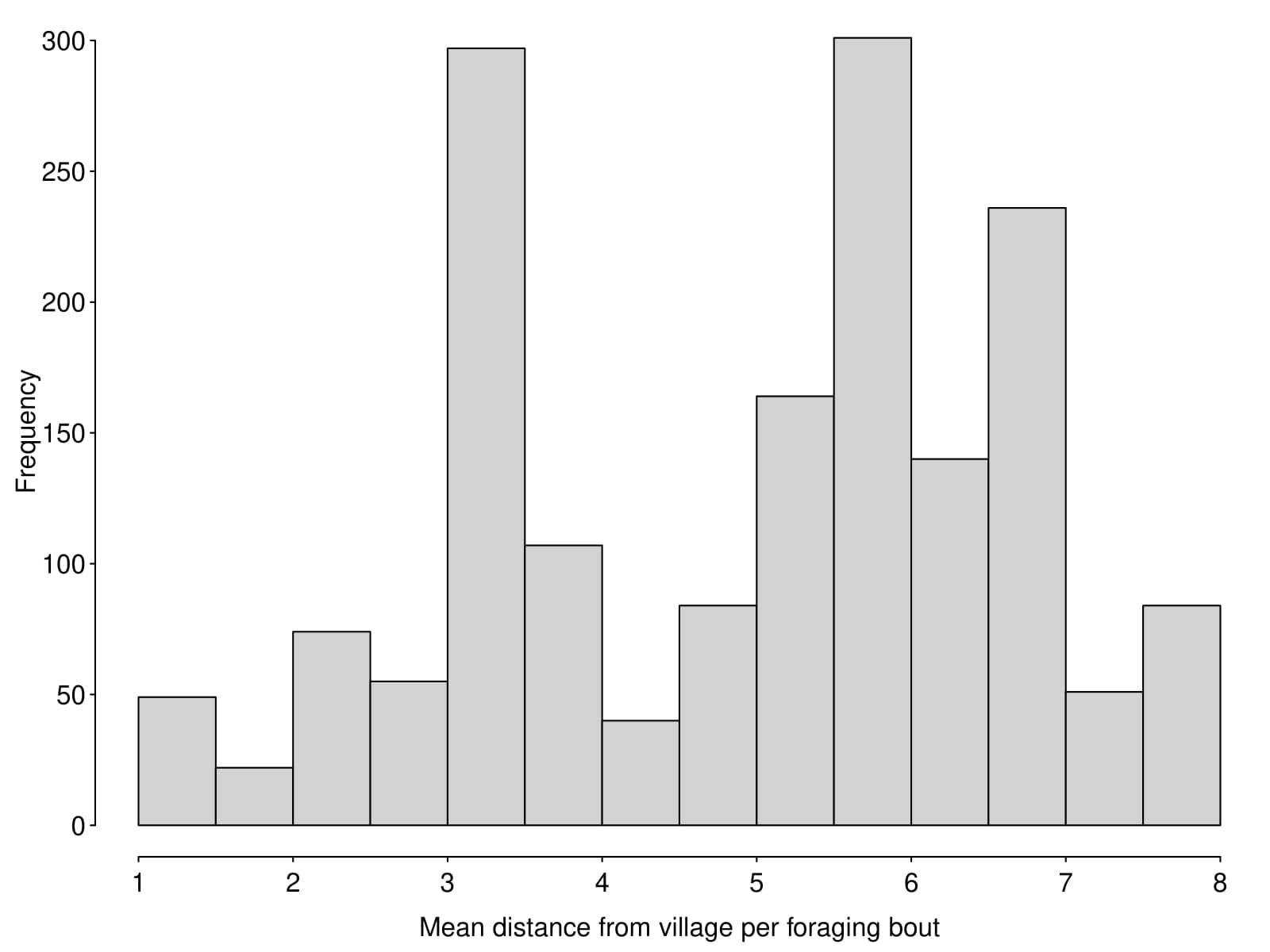

Supplement: Supplementary file 2 [file Data_Sheet_1.ZIP › Fig.S.2. Frequency distribution of all model variables. (D) mean distance from village per tuber searching and digging bout..jpg]

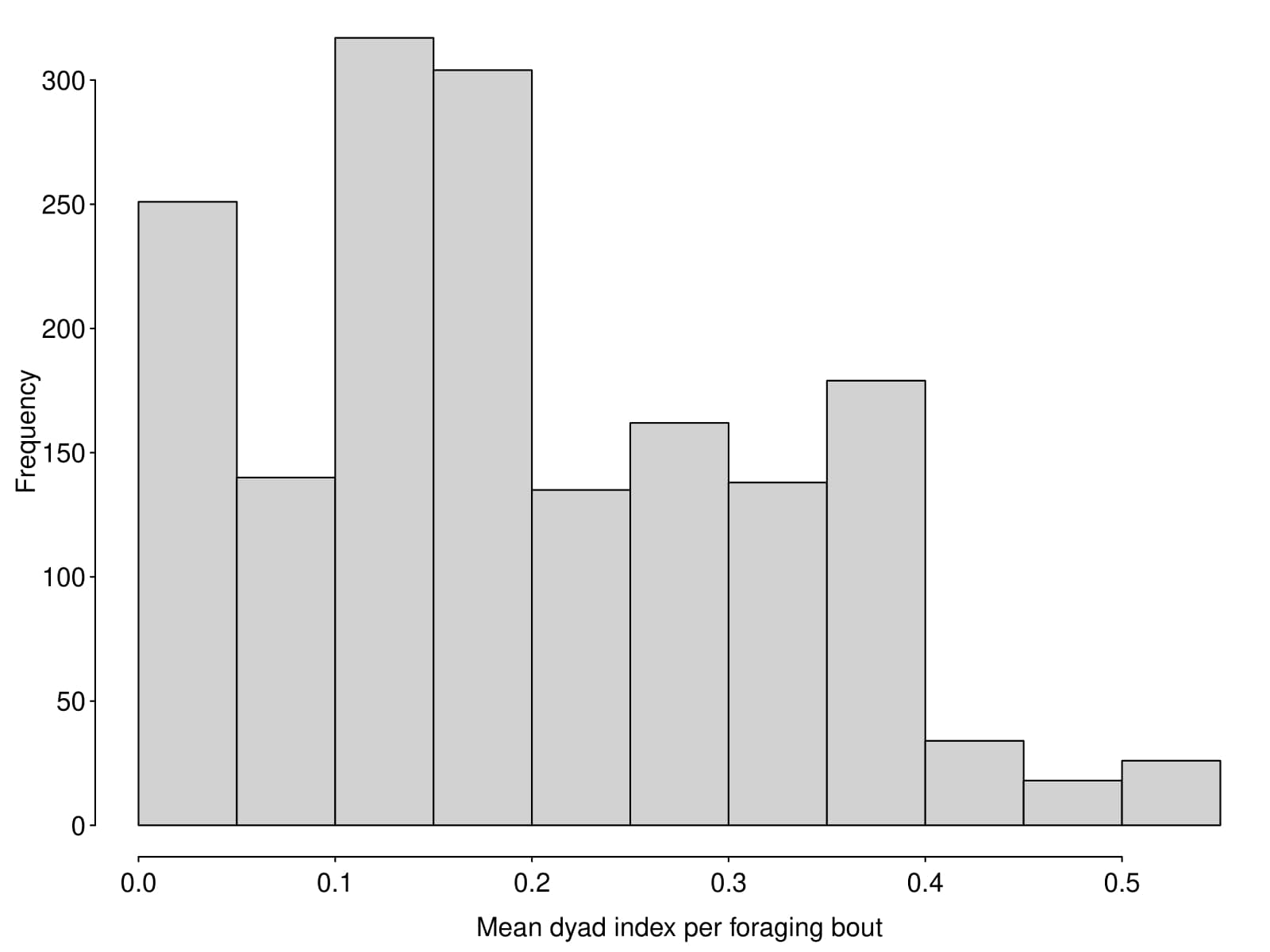

Supplement: Supplementary file 2 [file Data_Sheet_1.ZIP › Fig.S.2. Frequency distribution of all the model variables. (B) mean dyadic association index per tuber searching and digging bout.jpg]
